# EIF4A3 Promotes Muscle Atrophy and Aging by Inhibiting the FAK Pathway Through NEDD9 mRNA Destabilization

**DOI:** 10.1002/jcsm.70010

**Published:** 2025-07-10

**Authors:** Qian Li, Xiaohang Yin, Wensi Wan, Yi Zhou, Siqi Wang, Yuwei Yan, Jingying Chen, Xinyi Ren, Junli Gao, Yuying Chen, Yanan Zhang, Caiyue Cui, Emeli Chatterjee, Guoping Li, Ming Wu, Yan Zhang, Dongchao Lu, Tingting Yang, Yongjun Zheng, Jin Li

**Affiliations:** ^1^ Cardiac Regeneration and Ageing Lab, Institute of Geriatrics (Shanghai University), Affiliated Nantong Hospital of Shanghai University (The Sixth People's Hospital of Nantong), School of Medicine Shanghai University Nantong China; ^2^ Institute of Cardiovascular Sciences, Shanghai Engineering Research Center of Organ Repair, Joint International Research Laboratory of Biomaterials and Biotechnology in Organ Repair (Ministry of Education), School of Life Sciences Shanghai University Shanghai China; ^3^ Cardiovascular Division of the Massachusetts General Hospital and Harvard Medical School Boston Massachusetts USA; ^4^ Department of Orthopedics Shanghai Gongli Hospital Shanghai China; ^5^ School of Integrative Medicine Shanghai University of Traditional Chinese Medicine Shanghai China; ^6^ Department of Pain Medicine & Department of Rehabilitation Medicine Huadong Hospital Shanghai China

**Keywords:** EIF4A3, exon junction complex (EJC), FAK, muscle atrophy, NEDD9

## Abstract

**Background:**

Muscle atrophy has a poor prognosis, caused by various factors. Identifying a shared treatment target could address an unmet clinical need. The exon junction complex (EJC), a protein complex assembly that binds to RNA, facilitates post‐transcriptional regulation by participating in mRNA splicing, mRNA export, translation and nonsense‐mediated mRNA decay. This study aims to investigate the role of the EJC in muscle atrophy.

**Methods:**

Single‐cell transcriptome analysis and western blot were employed to analyse EJC expression in muscle atrophy. Overexpression of EJC helicase EIF4A3, as well as counteracting endogenous EIF4A3, was manipulated using lentiviral and adeno‐associated virus 8 (AAV8) at both in vitro and in vivo levels. Imaging, RT‐qPCR and immunoblot were utilized to identify phenotypes associated with muscle atrophy and aging. RNA‐seq, RIP‐seq, RT‐qPCR and RIP‐PCR were conducted to determine the targets of EIF4A3. A pharmacological approach that activates the downstream pathways in EIF4A3 knockdown muscle was employed to elucidate the molecular mechanisms of EIF4A3 in muscle atrophy.

**Results:**

The core RNA helicase of the EJC, EIF4A3, showed increased expression in atrophied muscles and aging human muscle (+150.43%, *n* = 5 in young and aged human, age: 26.20 ± 6.760 vs. 73.60 ± 5.030, *p* < 0.001) and aged mice muscle (+74.54% in male, +61.28% in female: *n* = 6 in young and aged mice in male/female, age: 3 months vs. 20 months, *p* < 0.001). In vitro studies demonstrated that EIF4A3 overexpression promoted muscle atrophy and aging in myotubes (*n* = 6, *p* < 0.05), while EIF4A3 inhibition mitigated these effects (*p* < 0.05). In vivo phenotypic analysis indicated that overexpression of EIF4A3 in skeletal muscle promoted muscle atrophy (*n* = 10, *p* < 0.05) including reduced grip strength (−42.36%, *p* < 0.001), running capacity (−21.24%, *p* < 0.001), contraction force (−19.62%, *p* < 0.001), muscle weight (gastrocnemius muscle: −15.75%; *p* < 0.001; tibialis anterior muscle: −9.50%, *p* < 0.01), myofiber size (−11.59%, *p* < 0.001) and worsened molecular phenotypes (all *p* < 0.05). Knockdown of EIF4A3 protected against muscle atrophy induced by various stimuli, including denervation (*n* = 10, *p* < 0.05), immobilization (*n* = 10, *p* < 0.05) and angiotensin II (*n* = 6–10, *p* < 0.05) in mice. Mechanistically, Neural Precursor Cell Expressed, Developmentally Down‐Regulated 9 (NEDD9) mRNA was identified as a direct target of EIF4A3. EIF4A3 promoted the decay of NEDD9 mRNA and inhibited the downstream focal adhesion kinase (FAK) and PI3K‐Akt pathway, promoting muscle atrophy. Pharmacological activation of the NEDD9‐FAK pathway abolished the pro‐atrophy effects of EIF4A3.

**Conclusions:**

Our findings shed significant light on the pivotal function of the EJC in muscle atrophy, revealing novel mechanisms that contribute to EJC‐related disorders. Providing a target for therapeutic interventions aimed at combating muscle atrophy.

## 1 Introduction

Muscle atrophy, characterized by a substantial decline in muscle mass and strength, is a multifaceted condition that not only accompanies the natural aging process but also manifests as a common comorbidity across a wide range of chronic diseases [1]. Skeletal muscle atrophy occurs frequently in a variety of diseases including heart failure, cancer, chronic kidney disease, chronic obstructive pulmonary disease (COPD), diabetes and in a wide range of conditions such as denervation, inactivity and aging. Patients with muscle atrophy confront an increased risk of falls, fractures, disabilities and various other adverse outcomes, such as increased rehospitalization [2]. Thus, muscle atrophy not only reduces the quality of life of patients but also increases the morbidity and mortality rate of diseases. The underlying mechanisms of muscle atrophy are complex, fundamentally centred on the balance between protein synthesis and degradation [3, 4]. Currently, there is no universally effective pharmacological treatment for muscle atrophy. While physical activity is recognized as the most potent non‐pharmacological intervention, it is important to note that the limitations of exercise training are such that it is only applicable to a select group of individuals [5, 6, 7]. Elucidating the complex mechanisms underlying muscle atrophy is crucial for the development of novel therapeutic approaches, underscoring the need for continued research in this area.

Recent transcriptomic and proteomic studies have significantly advanced our understanding of muscle atrophy's complex mechanisms and underlying pathophysiology. Notably, the critical finding of these omics investigations is the post‐transcriptional regulation of RNA could also participate in muscle atrophy. Especially the Exon Junction Complex (EJC), the RNA‐binding protein (RBP) complex, which assembled and associated with pre‐mRNA splicing, and mRNA export, translation and degradation, demonstrates the regulatory function in muscle atrophy [8, 9]. The central components of the EJC consist of four key proteins: RNA‐binding motif 8A (RBM8A), Mago homologue (MAGOH), eukaryotic initiation factor 4A3 (EIF4A3) and metastatic lymph node 51 (MLN51) [10]. The DEAD‐box RNA helicase, EIF4A3 (also known as DDX48), is a major RBP component involved in EJC assembly [11]. As a member of the helicase superfamily, EIF4A3 is involved in various RNA‐related processes, including ATP binding and hydrolysis, communication between ATP and RNA binding sites, and other features characteristic of an RNA helicase [12]. In the presence of ATP, EIF4A3 can form a stable heterodimer with MAGOH and RBM8A, which locks EIF4A3 onto mRNA about 20–24 nucleotides upstream of the exon–exon junction through tight, hydrophobic and conserved interaction interfaces, significantly impacting mRNA stability [13]. EIF4A3 exerts a significant regulatory impact in various diseases by modulating post‐transcriptional splicing and RNA stability [14, 15, 16]. Our previous study has shown that EIF4A3 levels were increased in many types of muscle atrophy [17]. However, the precise role of EIF4A3 in mediating muscle atrophy through the post‐transcriptional RNA processing pathway remains to be further investigated.

In this study, we observed increased EIF4A3 levels in muscle tissue under conditions of atrophy and aging. Our results reveal a significant correlation between EIF4A3 and muscle atrophy, suggesting that increased EIF4A3 levels play a role in the progression of muscle atrophy. Additionally, we have shown that EIF4A3 binds to neural precursor cell expressed developmentally down‐regulated protein 9 (NEDD9) mRNA, promoting the degradation of Nedd9 RNA, rather than influencing post‐transcriptional splicing. Collectively, our findings highlight the importance of post‐transcriptional regulation, particularly the role of EIF4A3, in the pathogenesis of muscle atrophy.

## 2 Materials and Methods

### 2.1 Animal Study

Male C57BL/6J mice (aged 6 to 8 weeks) were sourced from Charles River (Beijing, China) and were accommodated in the Specific Pathogen‐Free (SPF) experimental animal facility at the Animal Laboratory Center of Shanghai University. The mice were provided with standard pelleted feed and water ad libitum, all in an indoor environment maintained at 21°C–23°C, following a cycle of alternating 12‐h light and 12‐h darkness. All procedures involving mice were conducted strictly in accordance with the Guidelines for the Use and Care of Laboratory Animals in Biomedical Research published by the National Institutes of Health (No. 85‐23, Revised in 1996), and the experimental protocol was approved by the Ethics Committee of Shanghai University (approval number: ECSHU2022‐130). Muscle atrophy models were created following established protocols [17, 18, 19]. For the denervation muscle atrophy model (Den), the sciatic nerve was exposed and a 5‐mm segment was resected. For the immobilization model (Imo): the hind limbs of the mice were fixed at a 90° angle of flexion, and a 0.4 × 8 mm screw was inserted through the talus and calcaneus, securing it into the shaft of the tibia. For the Angiotensin II (Ang II) muscle atrophy model, the Ang II or PBS minipump was implanted into the back after anaesthesia. In addition, according to the method outlined in our prior study [20, 21], pair feeding of Ang II‐ and vehicle‐treated mice was conducted to compensate for the Ang II‐induced anorexia. After 1 week of treatment, samples were collected, and the muscle was isolated for testing.

### 2.2 Human Samples

The subjects were categorized into two groups: young and elderly, based on their age [17]. All protocols concerning human skeletal muscle samples were sanctioned by the Ethics Committee of Shanghai Gongli Hospital (GLYYls2024‐016) and all procedures conformed to the 1964 Helsinki declaration and its later amendments or comparable ethical standards. All individuals signed a written informed consent to participate.

Other methods were found online in the Supporting Information.

### 2.3 Statistical Analysis

The experimental data were analysed and plotted using GraphPad Prism 8.0. The comparison between two groups was performed using Student's *t*‐test. And multiple group comparisons were conducted using one‐way or two‐way ANOVA, followed by Dunnett's T3 or Bonferroni post hoc tests, depending on the homogeneity of variance assessed. Data were expressed as Mean ± SD.

## 3 Results

### 3.1 EIF4A3 Overexpression Facilitates Muscle Atrophy and Muscle Aging

**FIGURE 1:**
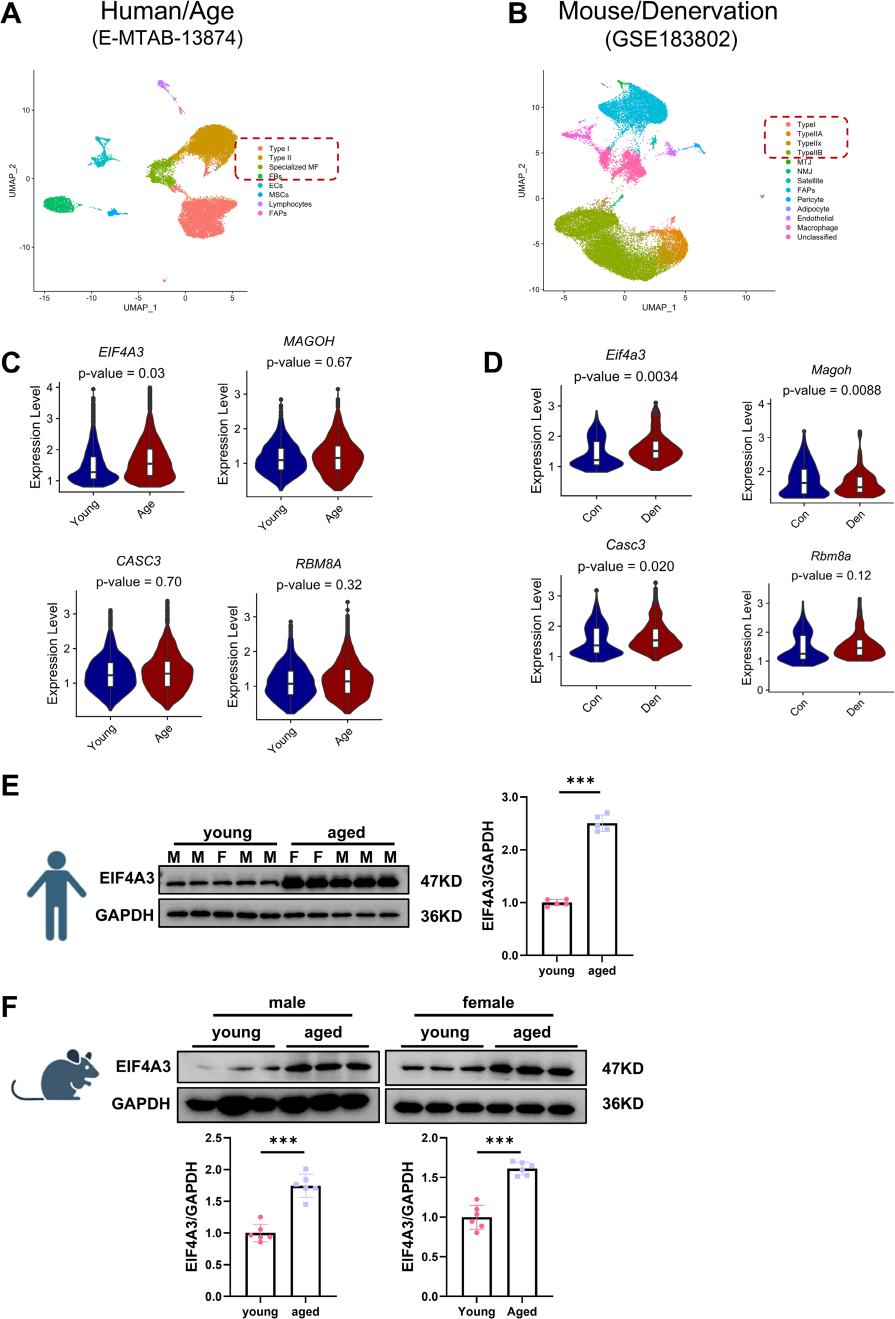
Expression of EIF4A3 in atrophic and aging muscle. (A) UMAP plot illustrating the transcriptomic landscape, consisting of eight major cell types. (B) scRNA‐seq analysis of mouse muscle tissue affected by atrophy. (C) *EIF4A3* expression was upregulated in aged muscle cells. (D) *Eif4a3* expression was upregulated in denervated muscle cells. (E)Western blot analysis of EIF4A3 expression levels in gastrocnemius muscle tissues of aged human (*n* = 5), M: male; F: female. (F) Western blot analysis of EIF4A3 expression levels in gastrocnemius muscle tissues of aged mice (male: *n* = 6; Female: *n* = 6). The comparison between two groups was performed using Student's *t*‐test. ***p < 0.001.

To analyse the EJC expression in muscle atrophy, we performed scRNA seqanalysis from the Gene Expression Omnibus (GEO) database (GSE183802) [22] as well as (E‐MTAB‐13874) [23] (Figure 1A,B). To assess the molecular changes associated with muscle atrophy, we performed differential gene expression analysis. Our results revealed a significant upregulation of EIF4A3 (human) and Eif4a3 (mouse) in muscle fibres, highlighting the essential role of EIF4A3 in the pathophysiology of muscle atrophy (Figure 1C,D).

Also, the core RNA helicase of the EJC, EIF4A3, was found to be associated with muscle atrophy and increased in muscle atrophy models in our previous work [17]. In addition, EIF4A3 was similarly found to be upregulated in muscle atrophy in female (Figure S1). Notably, in both skeletal muscle samples collected from aging mice in both male and female (20 months vs. 3 months) and aging human individuals (age: 73.60 ± 5.030 vs. 26.20 ± 6.760), EIF4A3 was significantly upregulated (Figure 1E,F).

**FIGURE 2:**
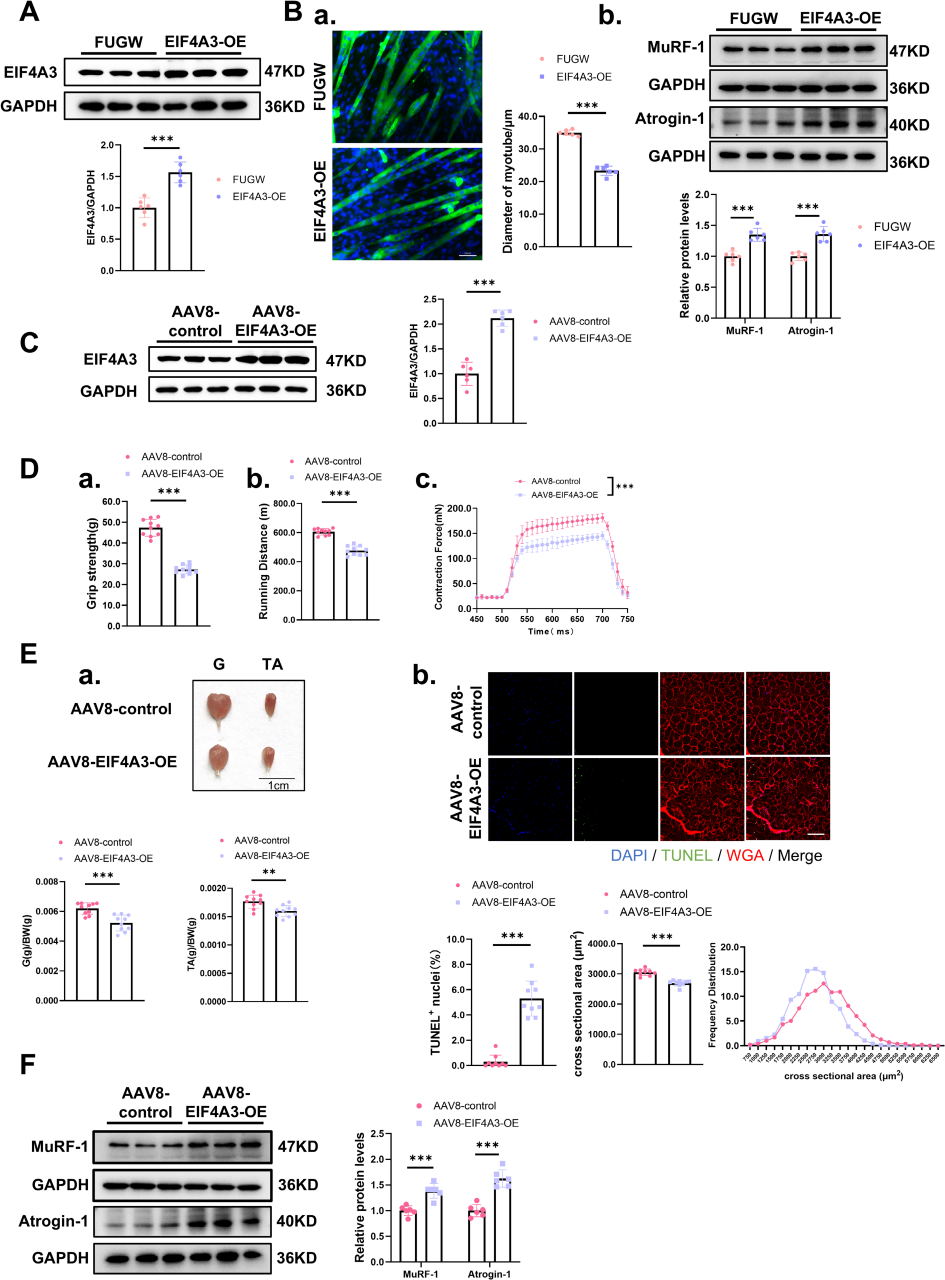
EIF4A3 triggers muscle atrophy in vitro and in vivo. (A) Expression levels of EIF4A3 in C2C12 myotubes transfected with EIF4A3 overexpression (EIF4A3‐OE) and controls (FUGW) lentivirus were evaluated by western blot (*n* = 6). (B) Representative images and statistical analysis of C2C12 myotubes transfected with EIF4A3‐OE and control lentivirus (*n* = 6), scale: 100 μm; Expression levels of MuRF‐1 and Atrogin‐1 protein levels in C2C12 myotube transfected with EIF4A3‐OE and controls lentivirus were evaluated by western blot (*n* = 6). (C) Analysis of EIF4A3 protein expression in gastrocnemius of mice injected with AAV8‐EIF4A3‐OE and AAV8‐control, by using Western Blot (*n* = 6). (D) Muscle function assay of mice injected with AAV8‐EIF4A3‐OE and AAV8‐control. (a) The grip strength (*n* = 10). (b) The running distance (*n* = 10). (c) EDL muscle contraction force (*n* = 6). (E) The size of muscle and myofiber of mice injected with AAV8‐EIF4A3‐OE and AAV8‐control. (a) The muscle morphology and weight (*n* = 10, scale: 1 cm); G: gastrocnemius; TA: tibialis anterior; BW: Body weight. (b) The cross‐sectional area and apoptotic level of muscle fibres (*n* = 8; Scale: 50 μm). (F) Analysis of MuRF‐1 and Atrogin‐1 protein levels in gastrocnemius of mice injected with AAV8‐EIF4A3‐OE and AAV8‐control evaluated by western blot (*n* = 6). The comparison between two groups was performed using Student's *t*‐test. ***p < 0.001.

Thus, to further clarify the function of EIF4A3 in muscle atrophy, exogenous overexpression of EIF4A3 was manipulated by lentiviral and adeno‐associated virus 8 (AAV8) in vitro and in vivo, respectively. To assess the function of EIF4A3 in muscle atrophy in vitro, the differentiated C2C12 myotubes were infected with EIF4A3 overexpression lentivirus for 2 days. The results showed that EIF4A3 overexpression did not affect the myogenic differentiation or the expression of fast‐ and slow‐type myofiber isoforms (Figure S2). Interestingly, lentiviral‐mediated EIF4A3 overexpression led to the reduction of myotube diameter compared with the control group (FUGW) (Figures 2A,B; S3A). In addition, EIF4A3 overexpression stimulated *Trim63*/MuRF‐1 and *Fbxo32*/Atrogin‐1 expressions and accelerated the ubiquitin‐proteasome system (UPS) process in muscle cells (Figures 2B; S3B–S3C). Consistently, both autophagy and apoptosis were induced following EIF4A3 overexpression (Figure S3D and S3E). Furthermore, global protein synthesis was reduced under EIF4A3 overexpression (Figure S3F). Notably, the upregulated expression of P53 and P16 was also observed under stimulation of EIF4A3, indicating accelerated myotube senescence (Figure S3G).

To obtain functional insights into the role of EIF4A3 in vivo, we produced AAV8‐EIF4A3 and AAV8‐control viral particles and administered them via injection into the skeletal muscles (gastrocnemius, tibialis anterior and extensor digitorum longus) of the right hind limb in adult male mice (Figure S4A). Following a 4‐week administration of AAV8, immunoblot analysis revealed that AAV8‐EIF4A3 treatment elicited a 3‐fold upregulation of *Eif4a3* mRNA levels, accompanied by a corresponding 2‐fold increase in EIF4A3 protein expression (Figure S4B; Figure 2C). The functionality of skeletal muscle was evaluated through assessments of exercise performance, grip strength measurements and the forces generated during tetanic contractions. Notably, skeletal muscle function was impaired in mice treated with AAV8‐EIF4A3 compared to control as observed by lower grip strength, running capacity and contraction force in overexpression group compared to the control group (Figure 2D). Meanwhile, the weight of skeletal muscles was reduced, and the myofiber diameter was diminished in these mice treated with AAV8‐EIF4A3 (Figure 2E). Furthermore, AAV8‐EIF4A3 treatment also resulted in a significant upregulation of the *Trim63*/MuRF‐1 and *Fbxo32*/Atrogin‐1 expressions expression in mouse skeletal muscle (Figures 2F, S4C). Additionally, the UPS, autophagy and apoptosis were activated, while protein synthesis was suppressed in mice treated with AAV8‐EIF4A3 (Figure S4D–S4G). Moreover, EIF4A3 overexpression did not alter the myofiber composition as observed by immunofluorescence staining, but a reduction of myofiber diameter observed in different types of myofibers (Figure S4H and S4I). Furthermore, the overexpression of EIF4A3 in skeletal muscle was found to be concomitant with significantly elevated expression levels of P53 and P16, suggesting an acceleration of muscle aging processes (Figure S4J).

**FIGURE 3:**
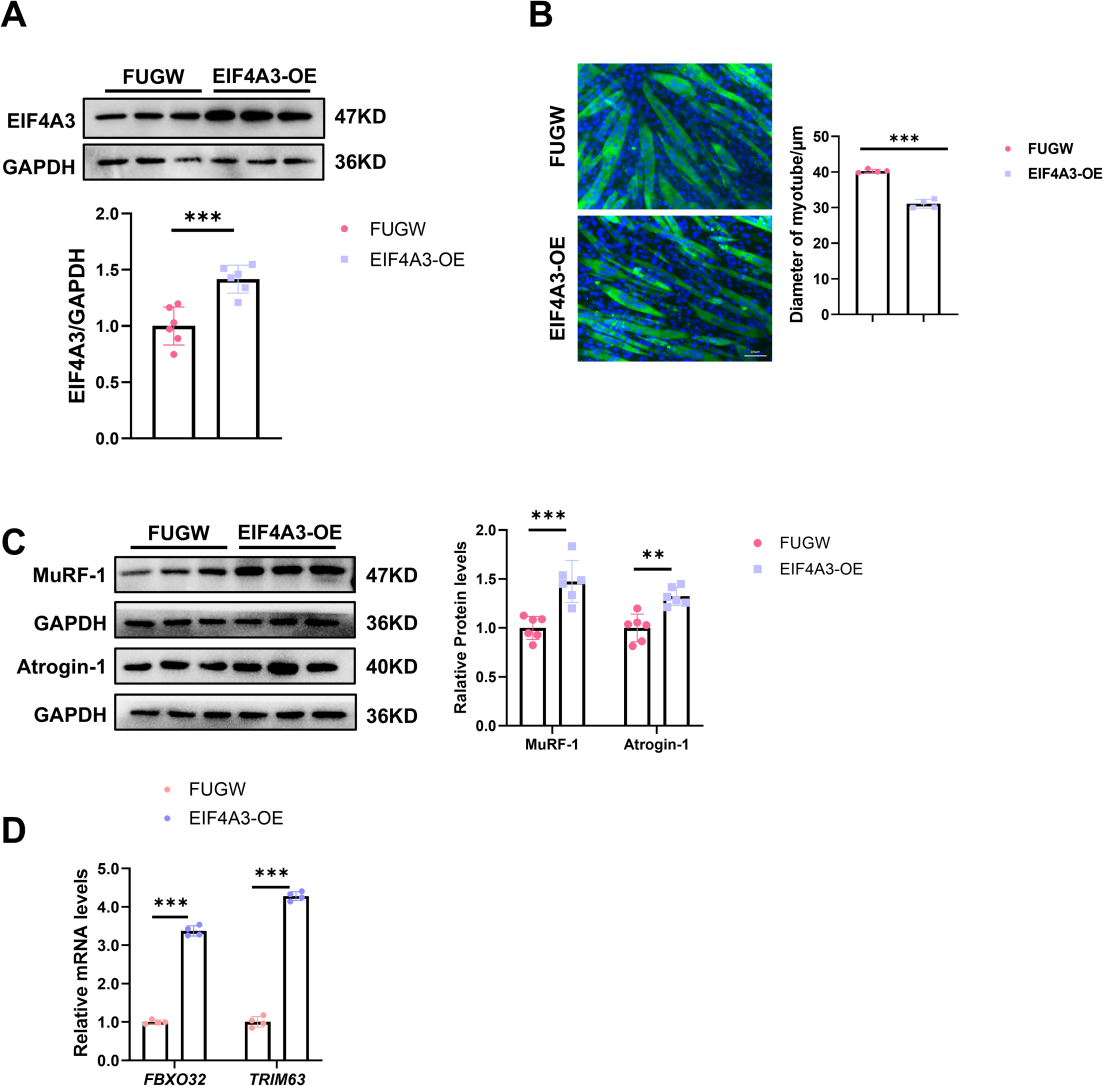
EIF4A3 triggers muscle atrophy in human myotube in vitro. (A) Expression levels of EIF4A3 in human myotubes transfected with EIF4A3 overexpression (EIF4A3‐OE) and controls (FUGW) lentivirus were evaluated by western blot (*n* = 6). (B) Representative images and statistical analysis of human myotubes transfected with EIF4A3‐OE and control lentivirus (*n* = 4), scale: 100 μm. (C) Expression levels of MuRF‐1 and Atrogin‐1 protein levels in human myotube transfected with EIF4A3‐OE and controls lentivirus were evaluated by western blot (*n* = 6). (D) Expression levels of *FBXO32* and *TRIM63* genes in human myotube transfected with EIF4A3‐OE and controls lentivirus were evaluated by RT‐qPCR (*n* = 4). The comparison between two groups was performed using Student's *t*‐test. **p < 0.01, ***p < 0.001.

To create a more realistic clinical scenario, we examined the function of EIF4A3 in human skeletal muscle myoblasts induced to myotube. The results also showed that EIF4A3 overexpression promoted muscle atrophy and did not affect the myogenic differentiation or the expression of fast‐ and slow‐type myofiber isoforms (Figures 3, S5).

Collectively, these findings indicate that an increased level of EIF4A3 in skeletal muscle leads to the activation of protein degradation pathways, the inhibition of protein synthesis and acceleration of muscle aging, suggesting that EIF4A3 is a key regulator in the initiation of muscle atrophy and senescence.

### 3.2 Inhibition of EIF4A3 Protects Against Muscle Atrophy

**FIGURE 4:**
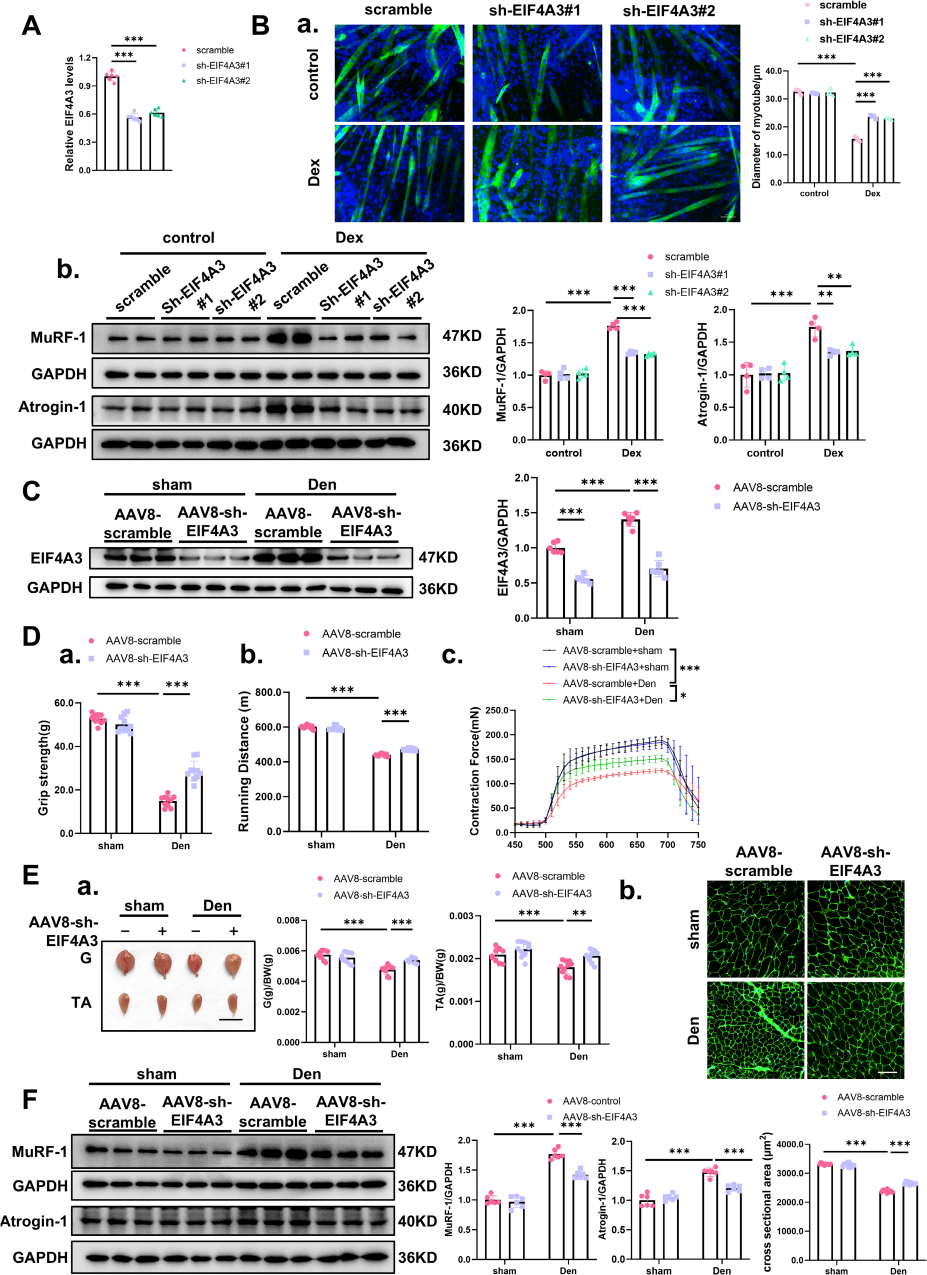
Inhibition of EIF4A3 expression prevents muscle atrophy in vitro and muscle atrophy caused by denervation in vivo. (A) EIF4A3 expression in C2C12 myotubes transfected with sh‐EIF4A3#1 and sh‐EIF4A3#2 lentivirus was analysed by RT‐qPCR (*n* = 6). (B) Immunofluorescence staining and quantification of the diameter of C2C12 myotubes transfected with sh‐EIF4A3#1 and sh‐EIF4A3#2 lentivirus in a dexamethasone (Dex)‐induced myotube atrophy model (*n* = 4; Scale: 100 μm); Expression of MuRF‐1 and Atrogin‐1 in C2C12 myotubes transfected with sh‐EIF4A3#1 and sh‐EIF4A3#2 lentivirus in Dex‐induced myotube atrophy model was analysed by western blot (*n* = 4). (C) Western blot analysis of protein expression levels of EIF4A3 in gastritis muscle of mice injected with AAV8‐sh‐EIF4A3 and AAV8‐scramble (*n* = 6). (D) Muscle function assay of mice injected with AAV8‐sh‐EIF4A3 and AAV8‐scramble. (a) Grip strength in right hind limb (*n* = 10). (b) Running distance (*n* = 10). (c) EDL muscle contraction force in the right hind limb (*n* = 6). (E) The size of muscle and myofiber of mice injected with AAV8‐sh‐EIF4A3 and AAV8‐scramble. (a) Representative images of morphology and body weight (G/BW, TA/BW) of gastrocnemius and tibialis anterior muscles (*n* = 10, scale: 1 cm). (b) Representative images and statistical analysis of muscle fibre cross‐sectional area (*n* = 8; Scale: 50 μm). (F) Expression levels of MuRF‐1 and Atrogin‐1 protein in gastrocnemius of mice injected with AAV8‐sh‐EIF4A3 and AAV8‐scramble were detected by western blot (*n* = 6). Multiple group comparisons were conducted using one‐way or two‐way ANOVA, followed by Dunnett's T3 or Bonferroni post hoc tests, depending on the homogeneity of variance assessed. **p < 0.01, ***p < 0.001.

To test whether counteracting endogenous EIF4A3 could reverse muscle atrophy, we designed two short hairpin RNAs (shRNAs), which specifically targeted the *Eif4a3* mRNA. Our results identified that two shRNAs can effectively degrade *Eif4a3* mRNA expression in myotube cells (Figure 4A). Silencing of EIF4A3 can reverse the dexamethasone (Dex), Tumour necrosis factor‐alpha (TNF‐α) and Angiotensin II (Ang II) induced muscle atrophy in vitro, as evidenced by the change of myotube size and the expression of atrophy‐related genes and proteins (Figures 4B; S6). Intriguingly, we found inhibition of EIF4A3 expression had no obvious impact on myogenic differentiation or fast‐ and slow‐type myofiber composition during dexamethasone treatment (Figure S7).

To evaluate the therapeutic potential of silencing EIF4A3 in vivo, we generated AAV8‐sh‐EIF4A3 and control AAV8‐control viral particles and administered them into the skeletal muscle. The treatment was followed by a 3‐week injection period and a subsequent 1‐week denervation (Den) surgery to establish a muscle atrophy model (Figure S8A). We observed that the AAV8‐sh‐EIF4A3 significantly suppressed the EIF4A3 expression in both sham and denervated mice (Figure S8B; Figure 4C). In this model, the inhibition of EIF4A3 significantly improved skeletal muscle function following Den surgery (Figure 4D). Meanwhile, we further demonstrated that AAV8‐sh‐EIF4A3 was able to attenuate muscle loss, increasing the size of myofibers and downregulate the muscle atrophy‐associated genes and proteins 1 week post Den surgery (Figures 4E,F, S8C). Moreover, AAV8‐sh‐EIF4A3 significantly activated protein synthesis in skeletal muscle (Figure S8D). In addition, EIF4A3 inhibition did not alter the myofiber composition as evaluated by immunofluorescence staining, but the rescue of myofiber diameter was observed in different types of myofibers (Figure S8E and S8F). These data suggest that the inhibition of EIF4A3 is an effective strategy for alleviating muscle atrophy induced by Den.

Given our observations that the upregulation of EIF4A3 appears to be a universal mediator in multiple types of muscle atrophy, we hypothesize that the silencing of EIF4A3 could represent a broadly applicable therapeutic strategy for various forms of muscle atrophy (Figures S9 and S10). Consistent with our hypothesis, in both immobilization ())Imo‐induced and Ang II‐induced muscle atrophy models, the administration of AAV8‐sh‐EIF4A3 significantly ameliorated muscle atrophy. This was demonstrated by the enhancement of skeletal muscle function, an increase in muscle weight and myofiber size, a decrease in the expression levels of several muscle atrophy marker proteins, and the stimulation of protein synthesis (Figures S9 and S10).

Collectively, our studies demonstrate that silencing EIF4A3 expression via shRNAs effectively mitigates muscle atrophy through multifaceted mechanisms, including enhanced muscle function, increased myofiber cross‐sectional area, decreased expression of atrophy‐related proteins and augmented protein synthesis. These findings underscore EIF4A3 as a promising therapeutic target for the treatment of muscle atrophy disorders.

### 3.3 EIF4A3 Promotes NEDD9 mRNA Decay in Muscle Atrophy

**FIGURE 5:**
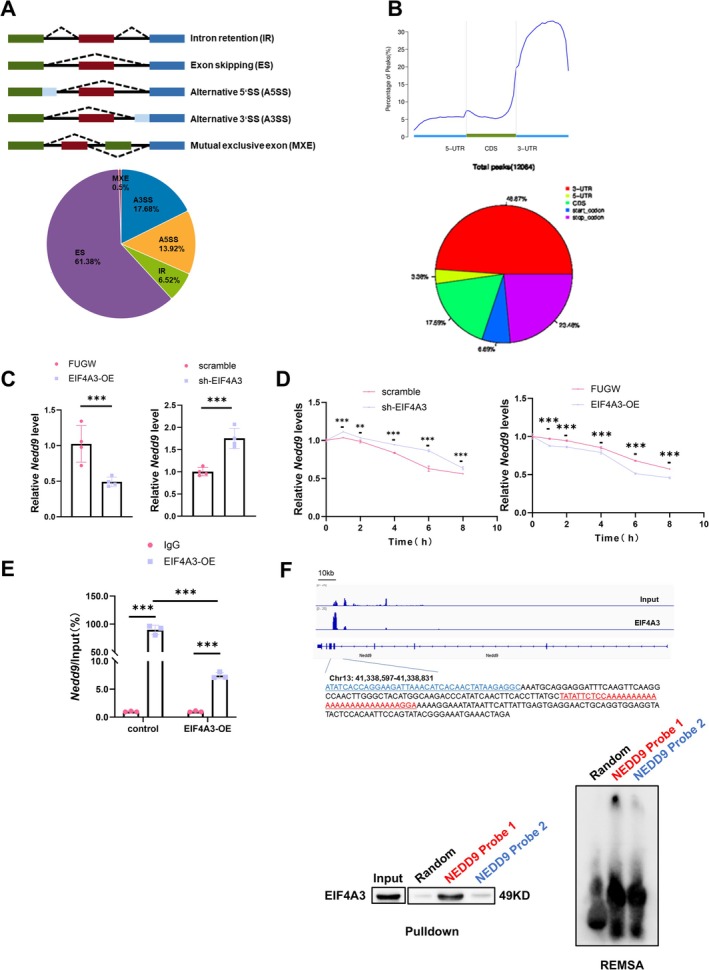
EIF4A3 plays a crucial role in NEDD9 mRNA decay, not alternative splicing. (A) Pie charts showing the percentage of different ASEs in EIF4A3 overexpression and a schematic diagram of alternative splicing types. ES, skipped exon; IR, retained intron; A3SS, alternative 3′ splice site; A5SS, alternative 5′ splice site; MXE, mutually exclusive exon. (B) Line charts and pie charts show EIF4A3 enrichment areas. (C)The expression level of *Nedd9* mRNA in C2C12 myotubes transfected with EIF4A3‐OE lentivirus sh‐EIF4A3 lentivirus group was detected by RT‐qPCR (*n* = 4). (D) The stability of *Nedd9* mRNA in C2C12 myotubes treated with ActD and sh‐EIF4A3 lentivirus EIF4A3‐OE lentivirus was determined by RT‐qPCR (*n* = 4). (E) RIP assay to analyse the enrichment of *Nedd9* mRNA by EIF4A3 antibody (*n* = 3). (F) RNA pulldown and REMSA was used to detect the binding sites of EIF4A3 and NEDD9 mRNA. The comparison between two groups was performed using Student's *t*‐test (C and D). Multiple group comparisons were conducted using one‐way or two‐way ANOVA, followed by Dunnett's T3 or Bonferroni post hoc tests, depending on the homogeneity of variance assessed (E). **p < 0.01, ***p < 0.001.

To explore the molecular impact of EIF4A3 in muscle atrophy, we conducted RNA‐seq on skeletal muscles from mice treated with AAV8‐control and AAV8‐EIF4A3. We observed that over 500 genes were dysregulated by EIF4A3 overexpression. Given that EIF4A3 is the core unit of EJC, we initially hypothesized that EIF4A3 may regulate RNA alternative splicing during muscle atrophy. To determine the impact of EIF4A3 on alternative splicing, we employed replicate multivariate analyses of transcript splicing (rMATS) on the RNA‐seq data, identifying 1595 differential alternative splicing events (ASEs) across 1124 genes (FDR < 0.05 and PS1 = 0.1). The predominant ASE was skipped exon (SE), constituting 61.38% of all events, followed by alternative 3′ splice site (A3SS, 17.68%), alternative 5′ splice site (A5SS, 13.92%), retained intron (RI, 6.52%) and mutually exclusive exon (MXE, 0.5%) (Figures 5A; S11A). Gene set enrichment analysis (GSEA) indicated significant enrichment of top molecular pathways involved in protein binding, RNA binding, ubiquitin–protein transferase activity, and ubiquitin protein ligase activity (Figure S11B). However, the majority of genes dysregulated by EIF4A3 overexpression were not associated with ASEs (Figure S11C). These data suggest that EIF4A3 does not regulate alternative splicing; instead, they imply that EIF4A3 may exert an unrecognized function in the pathogenesis of muscle atrophy.

To elucidate the molecular mechanisms of EIF4A3 in muscle atrophy, we conducted RIP‐seq for EIF4A3. Interestingly, our analysis revealed a high density of mapped reads in the 3′‐untranslated regions (3′‐UTRs), which are known to harbour regulatory elements that govern mRNA stability [24, 25] (Figure 5B). Thus, we further hypothesize that EIF4A3 regulates RNA stability rather than RNA alternative splicing during muscle atrophy. To elucidate the specific RNA molecules regulated by EIF4A3, we performed an integrative analysis combining RIP‐seq data with transcriptome profiles derived from EIF4A3‐modulated muscle tissue. This comprehensive approach identified 74 differentially expressed genes (DEGs) that were directly bound by EIF4A3, with the predominant majority (59/74) exhibiting downregulated expression (Figure S11D).

To screen the target RNAs of EIF4A3 in muscle atrophy, we employed RT‐qPCR to assess gene expression changes (Figure S11E). Notably, we discovered that EIF4A3 negatively regulated *Nedd9* mRNA expression in skeletal muscle (Figure 5C). Furthermore, we observed a reduction in *Nedd9* expression within muscle atrophy models (Figure S11F–S11H). Furthermore, we also determined that EIF4A3 influenced the mRNA stability of *Nedd9* by promoting its degradation (Figure 5D). RIP‐qPCR in C2C12 myotube cells confirmed the interaction between EIF4A3 and *Nedd9* mRNA, and EIF4A3 overexpression could significantly reduce the binding interaction between EIF4A3 and *Nedd9* mRNA (Figure 5E). Notably, our RIP‐seq analysis revealed that EIF4A3 occupies the 3′‐untranslated region (3′‐UTR) within the *Nedd9* mRNA. To validate this interaction, we employed RNA pulldown and REMSA, which confirmed the direct association between EIF4A3 and the *Nedd9* mRNA 3′‐UTR (Figure 5F). Additionally, we found no effect of EIF4A3 on *Nedd9* mRNA transport (Figure S11I).

To verify whether EIF4A3 promotes muscle atrophy by mediating NEDD9, we performed functional rescue experiments. Firstly, we found knockdown NEDD9 led to a smaller myotube and an enhanced expression of *Trim63*/MuRF‐1 and *Fbxo32*/Atrogin‐1 (Figure S12A–S12D). Then, we further found that NEDD9 overexpression could alleviate the muscle atrophy caused by EIF4A3 overexpression (Figure S12E–S12H).

Collection, these data suggest that EIF4A3 facilitates muscle atrophy via binding the NEDD9 mRNA and promoting RNA degradation.

### 3.4 EIF4A3/NEDD9 Inactivates FAK Pathway in Muscle Atrophy

**FIGURE 6:**
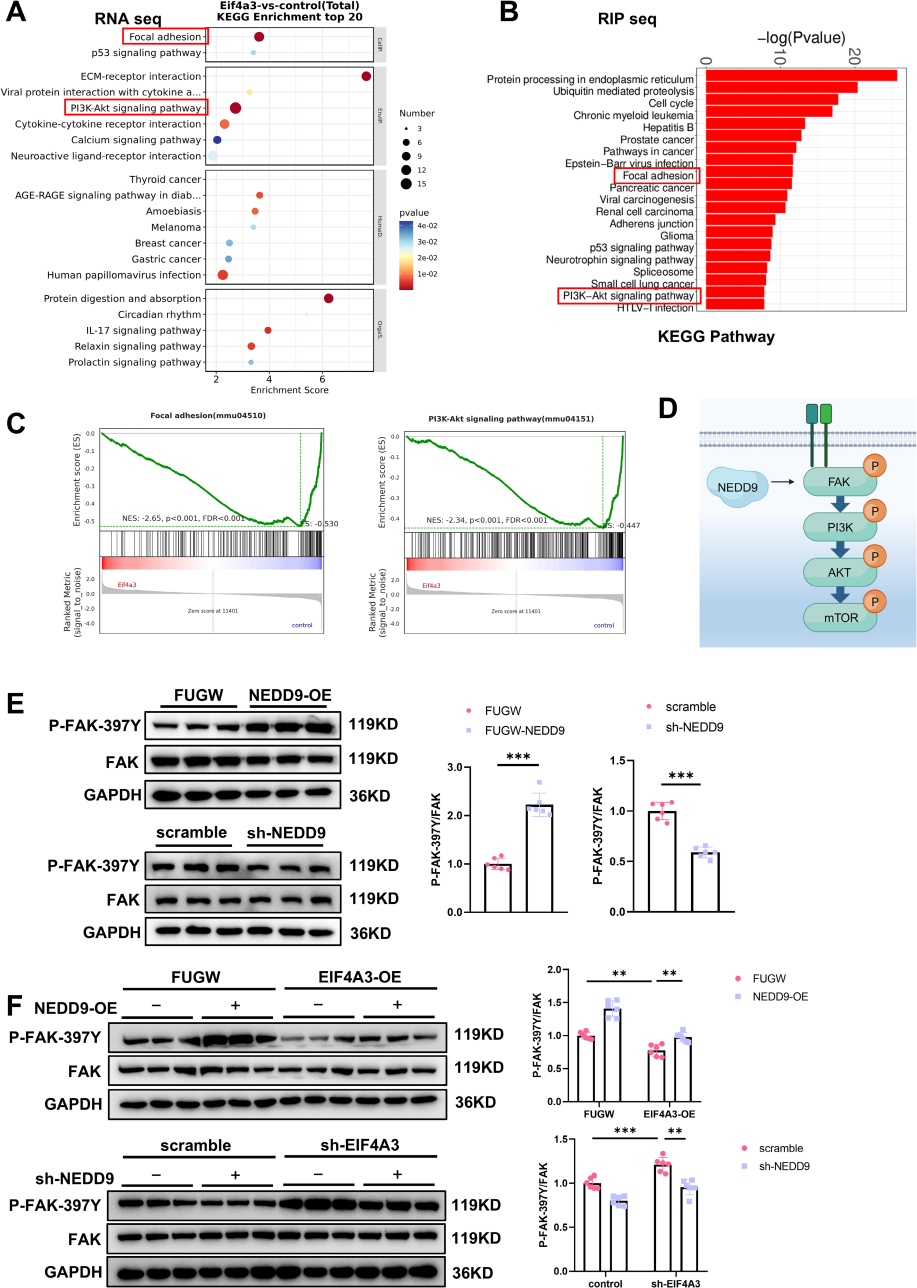
EIF4A3 mediates muscle atrophy by inhibition NEDD9‐FAK signalling pathway. (A) RNA seq was analysed for KEGG Enrichment, and (B) RIP seq was analysed for KEGG Pathway. (C) GSEA enrichment pathway analysis. (D) Model of signalling NEDD9‐FAK‐PI3K‐Akt‐mTOR pathway. NEDD9 binding to FAK, and resulting in the phosphorylation of FAK (397Y) followed by phosphorylation of PI3K, AKT and mTOR. (E)Western blot analysis for the FAK pathway in C2C12 myotubes transfected with NEDD9‐OE lentivirus (*n* = 6), sh‐NEDD9 lentivirus (*n* = 6). (F) Western blot analysis of FAK pathway in C2C12 myotubes transfected with EIF4A3‐OE and NEDD9‐OE lentivirus (*n* = 6), sh‐EIF4A3 and sh‐NEDD9 lentivirus (*n* = 6). The comparison between two groups was performed using Student's *t*‐test (E). Multiple group comparisons were conducted using one‐way or two‐way ANOVA, followed by Dunnett's T3 or Bonferroni post hoc tests, depending on the homogeneity of variance assessed (F). **p < 0.01, ***p < 0.001.

To elucidate the regulatory role of the EIF4A3/NEDD9 axis in muscle atrophy, we conducted Kyoto Encyclopedia of Genes and Genomes (KEGG) enrichment analyses (Figure 6A,B). The pathway enrichment analysis suggested that these dysregulated genes are implicated in the regulation of the Focal adhesion and PI3K‐Akt signalling pathways. Specifically, the expression of EIF4A3 was found to be inversely correlated with the activity of these pathways (Figure 6C,D). Furthermore, our analysis also implicated the involvement of additional pathways, including protein digestion and absorption, as well as ubiquitin‐mediated proteolysis. These findings are consistent with the characteristic molecular features of muscle atrophy (Figure 6A,B).

Notably, NEDD9 is a recognized binding partner and activator of FAK, enhancing its phosphorylation at Tyr397 (p‐FAK‐397Y). This interaction has been implicated in modulating cellular adhesion dynamics [26, 27]. To confirm this interaction between NEDD9 and FAK in muscle tissue, we observed that NEDD9 overexpression enhanced p‐FAK‐397Y levels, whereas NEDD9 knockdown had the opposite effect (Figure 6E). Importantly, p‐FAK‐397Y protein level was significantly downregulated in aging human muscle, aging mice muscle, atrophic muscle and atrophic myotube (Figure S13A–S13D). In addition, NEDD9 mRNA was also downregulated in aging muscle (Figure S13E and S13F). Furthermore, we found that EIF4A3 could reduce p‐FAK‐397Y levels by suppressing NEDD9 expression (Figure 6F). Collectively, our observations suggest that EIF4A3 may contribute to the development of muscle atrophy through the inhibition of the NEDD9‐FAK signalling axis. Conversely, these findings imply that activation of the NEDD9‐FAK signalling pathway may represent a potential therapeutic strategy for the treatment of muscle atrophy.

To provide additional evidence in support of our hypothesis, we choose vorinostat (suberoylanilide hydroxamic acid, SAHA), which activate the NEDD9‐FAK signalling pathway [28]. Consistent with our predictions, treatment of EIF4A3‐overexpressing myotubes with SAHA resulted in the activation of the NEDD9‐FAK signalling pathway, both in baseline or EIF4A3 overexpressed myotube (Figure S14A). Interestingly, we found that SAHA could increase myotube size and decreased the expression of *Trim63*/MuRF‐1 and *Fbxo32*/Atrogin‐1 (Figure S14B–S14D). These findings suggest that activating the NEDD9‐FAK signalling pathway with SAHA may alleviate myotube atrophy.

**FIGURE 7:**
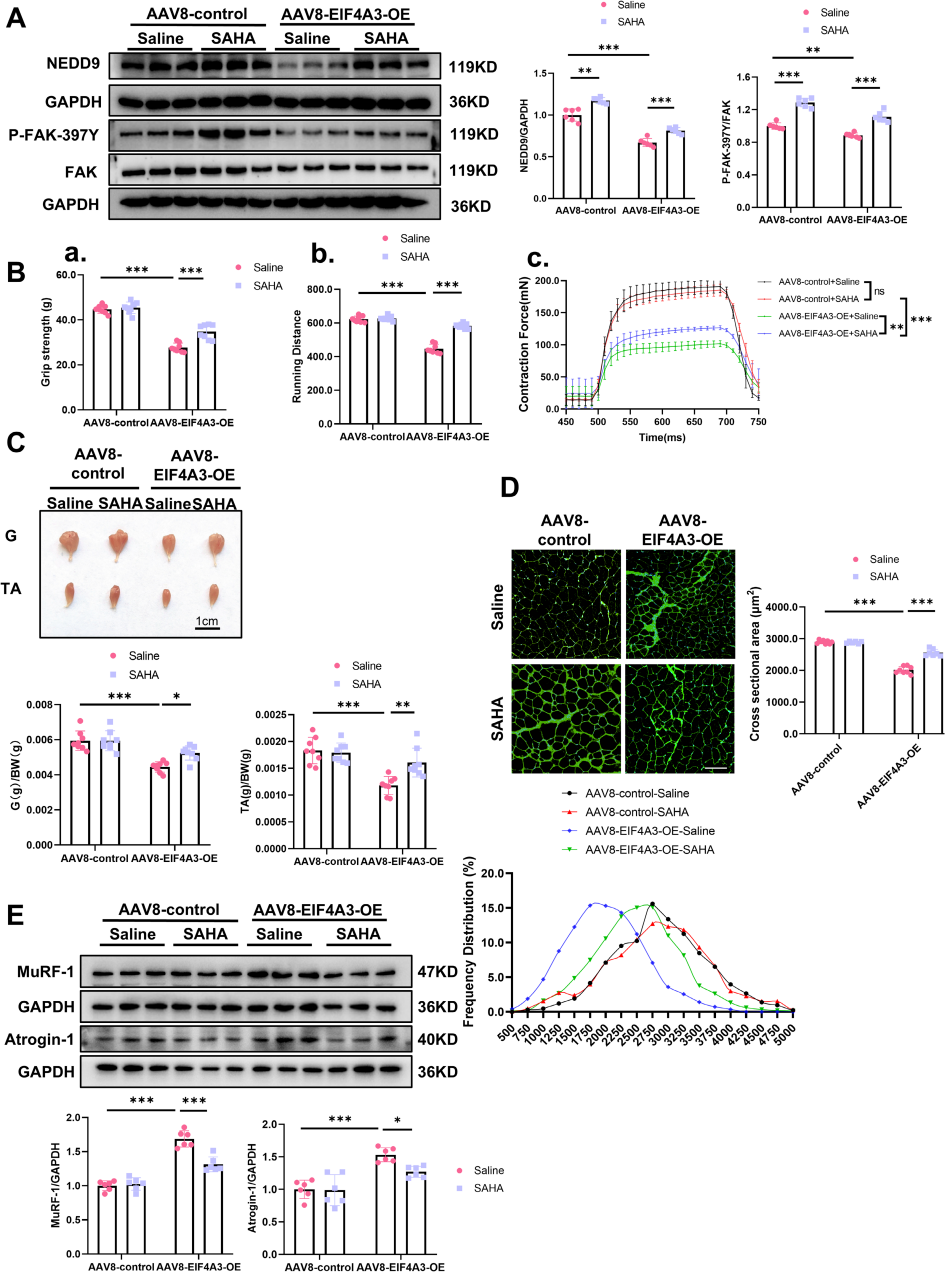
SAHA stimulation of NEDD9‐FAK signalling pathway alleviates muscle atrophy induced by EIF4A3 overexpression. (A) Western blot analysis of NEDD9 protein expression level and FAK pathway expression in gastrocnemius of mice injected with AAV8‐EIF4A3‐OE and SAHA (*n* = 6). (B) Muscle function assay of in mice injected with AAV8‐EIF4A3‐OE and SAHA. (a) Grip strength of right hind limb muscles (*n* = 8). b. Running distance (*n* = 8). (c) EDL muscle contraction force (*n* = 6). (C) Representative images of gastrocnemius and tibialis anterior muscle morphology and specific weight (G/BW, TA/BW) of gastrocnemius in mice injected with AAV8‐EIF4A3‐OE and SAHA (*n* = 10, scale: 1 cm). (D) Representative images and statistical analysis of muscle fibre cross‐sectional area in mice injected with AAV8‐EIF4A3‐OE and SAHA (*n* = 8; Size: 50 μm). (E) Expression levels of MuRF‐1 and Atrogin‐1 proteins in gastrocnemius of mice injected with AAV8‐EIF4A3‐OE and SAHA were detected by western blot (*n* = 6). Multiple group comparisons were conducted using one‐way or two‐way ANOVA, followed by Dunnett's T3 or Bonferroni post hoc tests, depending on the homogeneity of variance assessed. *p < 0.05, **p < 0.01, ***p < 0.001.

Following the injection of AAV8‐EIF4A3 for 1 week, mice were intraperitoneally administered SAHA at a dosage of 50 mg/kg/day for an additional 4 weeks (Figure S15A). Our results demonstrated that SAHA could stimulate the NEDD9‐FAK signalling pathway in both baseline and EIF4A3‐overexpressed muscle conditions (Figure 7A). Further findings indicated that while EIF4A3 induced muscle atrophy, as previously shown, SAHA mitigated these effects by enhancing muscle function, increasing muscle and myofiber size and promoting protein synthesis (Figures 7B–E; S15B–S15C). In addition, SAHA did not alter the myofiber composition as examined by immunofluorescence staining, but the rescue of myofiber diameter in different types of myofibers was induced by EIF4A3 overexpression (Figure S15D and S15E). These results suggest that EIF4A3 mediates muscle atrophy by inhibiting NEDD9‐FAK signalling pathway.

**FIGURE 8:**
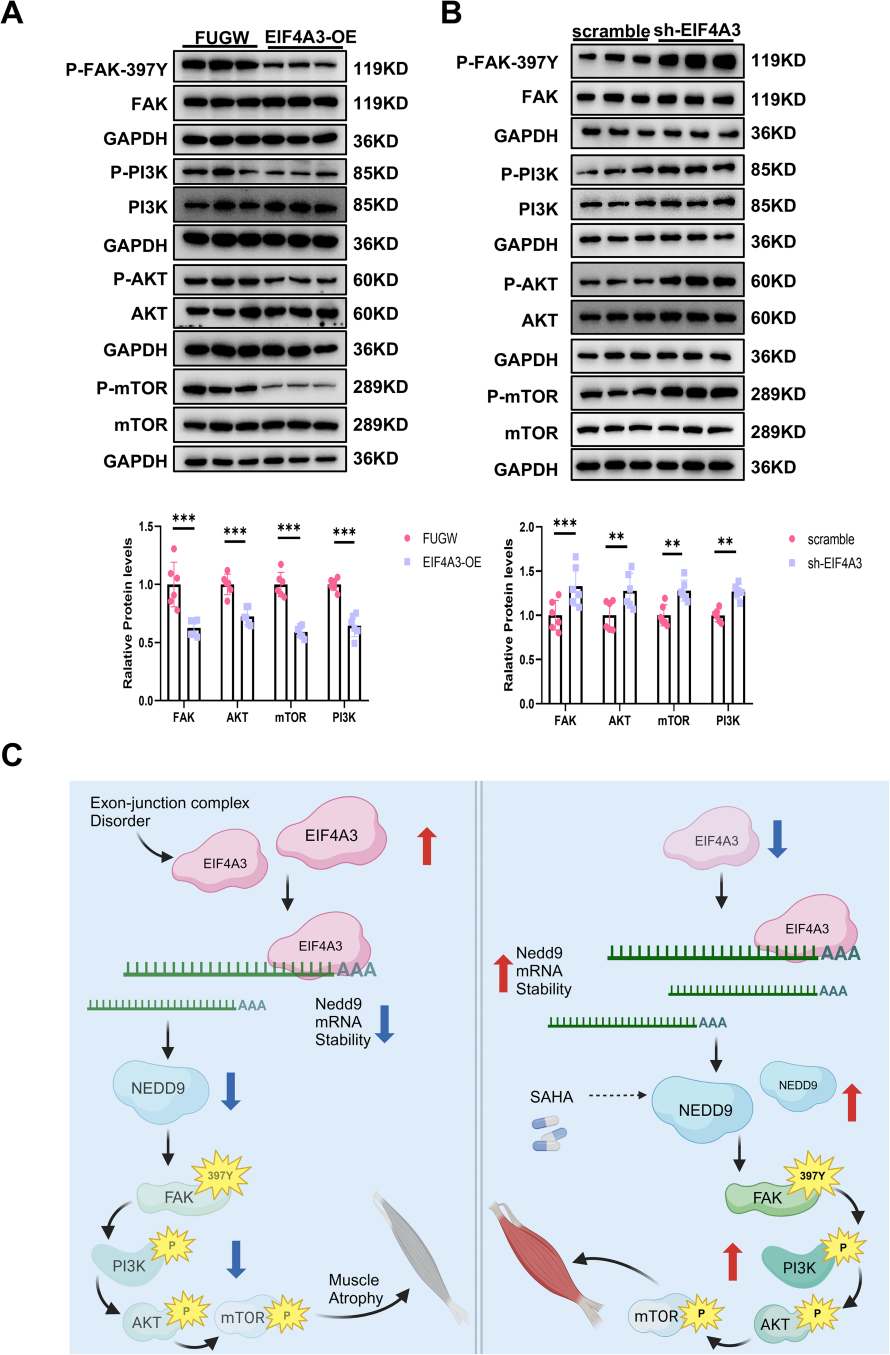
EIF4A3 inhibits FAK‐PI3K‐AKT‐mTOR pathway. Western Blot analysis of FAK‐PI3K‐AKT‐mTOR pathway expression in C2C12 myotubes treated with (A) EIF4A3‐OE (*n* = 6) and (B) sh‐EIF4A3 (*n* = 6). (C) Illustration of the mechanisms of EJC in muscle atrophy. The figure is created with BioRender (Biorender.com). The comparison between two groups was performed using Student's *t*‐test. **p < 0.01, ***p < 0.001.

Given that the kinase function of FAK is essential for the reduction of cells apoptosis, promoting cellular survival and enhancing protein synthesis [29, 30], we further evaluate the p‐PI3K, p‐AKT as well as p‐mTOR level in the context of EIF4A3 overexpression as well as silencing. Our results demonstrated that EIF4A3 suppresses P‐FAK‐397Y, consequently inactivating the PI3K‐Akt‐mTOR pathway, while the knockdown of EIF4A3 elicited the converse effect (Figure 8A).

## 4 Discussion

Our work reveals that EIF4A3 is a trigger in muscle atrophy and muscle aging (Figure 8B). As an RNA binding protein, EIF4A3 binds to NEDD9 mRNA and promotes RNA decay. Consequently, EIF4A3 directly suppresses NEDD9‐FAK and PI3K‐Akt pathways, sequentially. Silencing EIF4A3 levels or stimulating the NEDD9‐FAK signalling pathway could potentially serve as therapeutic strategy for muscle atrophy.

RNA post‐transcriptional regulation is a key mechanism in gene expression that plays a crucial part in a variety of biological processes. Specifically, alternative splicing, mRNA stability and localization are recognized as pivotal points for post‐transcriptional regulation. RBPs comprise a functionally diverse group of regulators that play indispensable roles in modulating post‐transcriptional processes. EIF4A3, a central helicase component of the EJC, has been extensively characterized as a multifunctional regulator of RNA metabolism, influencing RNA splicing, mRNA transport and nonsense‐mediated mRNA decay. In some cancer cell, EIF4A3 influences the generation of circular RNAs through alternative splicing, promoting the tumorigenesis [31]. In skeletal muscle system, EIF4A3 knockdown impairs human myoblasts (C25CL48) differentiation via EJC [32]. In our study, we did not observe any changes in mice myoblast differentiation when EIF4A3 was either overexpressed or inhibited during dexamethasone treatment. Altering the expression of EIF4A3 was performed on the mature myotube cell level, which may lead the difference. In addition, EIF4A3 did not alter the myofiber composition or the expression of fast‐ and slow‐type myofiber isoforms in muscle. Our research has demonstrated that EIF4A3 is upregulated in multiple forms of muscle atrophy and plays a regulatory role in muscle atrophy. Nevertheless, the precise molecular mechanisms underlying EIF4A3's involvement in muscle atrophy remain to be elucidated.

In this work, we further found that EIF4A3 was also increased in aging human and mice muscle. Our data proved that EIF4A3 mainly regulated nonsense‐mediated mRNA decay by binding with 3′‐UTR regions of mRNA, rather than alternative RNA splicing or mRNA transport. Thus, some EIF4A3‐selective inhibitor that suppresses nonsense‐mediated mRNA decay may represent a new treatment method [33, 34]. Through integrated analysis of RNA‐Seq and RIP‐Seq, we identified NEDD9 as a direct target molecule of EIF4A3. Furthermore, our findings suggest that EIF4A3's function is not solely limited to its role within the EJC, highlighting the importance of exploring EJC‐independent functions of EIF4A3 in future investigations [16, 35, 36].

The second important discovery is the role of the NEDD9‐FAK pathway in muscle atrophy. NEDD9, also named as CasL and HEF1, is a multi‐domain scaffolding protein involved in cell signalling by binding and activating SRC, FAK and Aurora‐A kinase. NEDD9 also participates in multiple cellular processes, such as cell proliferation, DNA damage response, apoptosis, migration, adhesion, invasion and development. However, there is no relevant research on the skeletal muscle system. Our study reveals a novel link between NEDD9 downregulation and muscle atrophy. NEDD9 knockdown resulted in reduced myotube size and increased expression of E3 ubiquitin ligases, Atrogin‐1 and MuRF‐1. These data imply the correlation between NEDD9 and muscle atrophy. Interestingly, the focal adhesion pathway was significantly enriched in the RNA‐Seq and RIP‐Seq analyses of EIF4A3, exhibiting a negative correlation with muscle atrophy. In addition, NEDD9 promoted P‐FAK‐397Y, while NEDD9 inhibition reduced this effect. Given that FAK is a well‐established downstream target of NEDD9, our findings suggest that the NEDD9‐FAK pathway is negatively correlated with muscle atrophy. Based on these results, we propose that pharmacological activation of the NEDD9‐FAK pathway may represent a promising therapeutic strategy for mitigating muscle atrophy.

The compound, SAHA (suberoylanilide hydroxamic acid), which has been reported to activate the NEDD9‐FAK signalling pathway [28, 37]. Consistently, we discovered that SAHA can activate the NEDD9‐FAK pathway and protect against muscle atrophy induced by EIF4A3, both in vitro and in vivo. Nevertheless, it is imperative to further evaluate the therapeutic potential of SAHA in the treatment of muscle atrophy.

In addition, we first reported that P‐FAK‐397Y was downregulated in atrophic muscle and aged muscle. Consistent with our results, P‐FAK‐397Y was also reduced in human vastus lateralis after of unloaded [38, 39]. In contrast, P‐FAK‐397Y content in muscle was increased after resistance training, which may modulate the muscle protein synthesis [40, 41]. In skeletal muscle, the role of FAK is pleiotropic in regulating in muscle homeostasis and muscle health, such as promoting myogenesis, muscle phenotype, costamere formation, muscle hypertrophy and glucose uptake [42]. FAK is upstream of PI3K‐Akt pathway. As expected, PI3K‐Akt pathway was enrichment in the RNA‐Seq and RIP‐Seq of EIF4A3, and positive correlation with muscle atrophy. We also found P‐FAK‐397Y was reduced in aged muscle and in a variety of muscle atrophy models.

Thus, the findings of the present study emphasized the potential roles of the EIF4A3‐NEDD9‐FAK pathway in aging and in muscle atrophy diseases.

## Conflicts of Interest

The authors declare no conflicts of interest.

## Online Supplementary Material

Additional supporting information may be found online in the Supporting Information section at the end of the article.

## Supporting information


**Figure S1.** Expression of EIF4A3 in atrophic muscle in female mice. Western blot analysis of EIF4A3 expression levels in gastrocnemius muscle tissues of denervation (Den)‐ (A), immobilization (Imo)‐ (B) and Angiotensin II (Ang II)‐ (C) induced muscle atrophy models in female mice (*n* = 4 per group). The comparison between two groups was performed using Student’s *t*‐test. The statistical results were represented by Mean ± SD. **p < 0.01, ***p < 0.001.
**Figure S2.** EIF4A3 overexpression does not affect the myogenic differentiation and fast‐ and slow‐type isoforms in mice myotube cells. (A) The myotube fusion index of C2C12 myotubes transfected with EIF4A3‐OE for 2 days (*n* = 4 per group). (B) Analysis of myogenic differentiation‐associated genes and fast‐ and slow‐type isoform‐associated genes in C2C12 myotubes transfected with EIF4A3‐OE and FUGW lentivirus were evaluated by RT‐qPCR (*n* = 6). (C and D) Western blot analysis of MyoD, fast or slow myosin heavy chain (MyHC) protein expression levels in C2C12 myotubes transfected with EIF4A3‐OE and control lentivirus (*n* = 6). The comparison between two groups was performed using Student’s *t*‐test. The statistical results were represented by Mean ± SD.
**Figure S3.** EIF4A3 promotes muscle atrophy and muscle aging in vitro. (A) Expression levels of *Eif4a3* in C2C12 myotubes transfected with EIF4A3 overexpression (EIF4A3‐OE) and controls (FUGW) lentivirus were evaluated by RT‐qPCR (*n* = 6). (B) Expression levels of *Fbxo32* and *Trim63* genes in C2C12 myotube transfected with EIF4A3‐OE and controls lentivirus were evaluated by RT‐qPCR (*n* = 6). (C) Western blot analysis of ubiquitin–protein expression in C2C12 myotubes transfected with EIF4A3‐OE and control lentivirus (*n* = 6). (D) Western blot analysis of P62 and LC3 protein expression levels in C2C12 myotubes transfected with EIF4A3‐OE and control lentivirus (*n* = 6). (E) Western blot analysis of caspase 3 and Bax/Bcl_2_ protein expression levels in C2C12 myotubes transfected with EIF4A3‐OE and control lentivirus (*n* = 6). (F) Western blot analysis of protein synthesis by anti‐Puromycin in C2C12 myotubes transfected with EIF4A3‐OE and control lentivirus (*n* = 6). (G) Western blot analysis of expression levels of aging marker proteins P53 and P16 in C2C12 myotubes transfected with EIF4A3‐OE and control lentivirus (*n* = 6). The comparison between two groups was performed using Student’s *t*‐test. The statistical results were represented by Mean ± SD. *p < 0.05, **p < 0.01, ***p < 0.001.
**Figure S4.** EIF4A3 induces muscle atrophy in vivo. (A) Schematic design of the experiment including the viral injection dose and the experimental time point. (B) Expression level of *Eif4a3* in gastrocnemius of mice injected with AAV8‐EIF4A3‐OE and AAV8‐control evaluated by RT‐qPCR (*n* = 10). (C) Analysis of *Fbxo32* and *Trim63* mRNA levels in gastrocnemius of mice injected with AAV8‐EIF4A3‐OE and AAV8‐control evaluated by RT‐qPCR (*n* = 10). (D) Western blot analysis of ubiquitin‐protein expression in gastrocnemius of mice injected with AAV8‐EIF4A3‐OE and AAV8‐ controls (*n* = 6). (E) Western blot analysis of P62 and LC3 protein expression levels in gastrocnemius of mice injected with AAV8‐EIF4A3‐OE and AAV8‐ controls (*n* = 6). (F) Western blot analysis of caspase3 and Bax/Bcl_2_ protein expression levels in gastrocnemius of mice injected with AAV8‐EIF4A3‐OE and AAV8‐control (*n* = 6). (G) Western blot analysis of protein synthesis by anti‐ Puromycin in muscle of mice injection with AAV8‐EIF4A3‐OE and AAV8‐ control (*n* = 6). (H) The types of gastrocnemius fibres in mice injected with AAV8‐EIF4A3‐OE and AAV8‐control were detected by immunofluorescence staining (*n* = 7 in AAV8‐control group, *n* = 8 in AAV8‐EIF4A3‐OE group). (I) Western blot analysis of fast or slow myosin heavy chain (MyHC) protein expression levels in muscle of mice injection with AAV8‐EIF4A3‐OE and AAV8‐ control (*n* = 6). (J) Western blot analysis of expression levels of aging marker proteins P53 and P16 in gastrocnemius of mice injected with AAV8‐EIF4A3‐OE and AAV8‐control (*n* = 6). The comparison between two groups was performed using Student’s *t*‐test. The statistical results were represented by Mean ± SD. *p < 0.05, **p < 0.01, ***p < 0.001.
**Figure S5.** EIF4A3 overexpression does not affect the myogenic differentiation and fast‐ and slow‐type isoforms in human myotube cells. (A) The myotube fusion index of human myotubes transfected with EIF4A3‐OE for 2 days (*n* = 4 per group). (B) Analysis of myogenic differentiation‐associated genes and fast‐ and slow‐type isoform‐associated genes in human myotubes transfected with EIF4A3‐OE and FUGW lentivirus were evaluated by RT‐qPCR (*n* = 4). (C and D) Western blot analysis of MyoD, fast or slow myosin heavy chain (MyHC) protein expression levels in human myotubes transfected with EIF4A3‐OE and control lentivirus (*n* = 6). The comparison between two groups was performed using Student’s t‐test. The statistical results were represented by Mean ± SD.
**Figure S6.** Inhibition of EIF4A3 expression prevents muscle atrophy in vitro. (A) Expression of *Fbxo32* and *Trim63* in C2C12 myotubes transfected with sh‐EIF4A3#1 and sh‐EIF4A3#2 lentivirus in Dex‐induced myotube atrophy model was analysed by RT‐qPCR (*n* = 4). (B) Immunofluorescence staining and quantification of C2C12 myotubes diameter transfected with sh‐EIF4A3#1 and sh‐EIF4A3#2 lentivirus in a TNF‐α‐induced muscular atrophy model (*n* = 4; Scale:100 μm). (C) *Fbxo32* and *Trim63* expression in C2C12 myotubes transfected with sh‐EIF4A3#1 and sh‐EIF4A3#2 lentivirus in a TNF‐α‐induced muscle atrophy model was analysed by RT‐qPCR (*n* = 4). (D) MuRF‐1 and Atrogin1 expression in C2C12 myotubes transfected with sh‐EIF4A3#1 and sh‐EIF4A3#2 lentivirus in a TNF‐α‐induced muscle atrophy model was analysed by western blot (*n* = 4). (E) Immunofluorescence staining and quantification of C2C12 myotubes diameter transfected with sh‐EIF4A3#1 and sh‐EIF4A3#2 lentivirus in Ang II‐induced muscle atrophy models. (*n* = 4; Scale: 100 μm). (F) *Fbxo32* and *Trim63* expression in C2C12 myotubes transfected with sh‐EIF4A3#1 and sh‐EIF4A3#2 lentivirus in a Ang II‐induced muscle atrophy model was analysed by RT‐qPCR (*n* = 4). (G) MuRF‐1 and Atrogin1 expression in C2C12 myotube transfected with sh‐EIF4A3#1 and sh‐EIF4A3#2 lentivirus in Ang II‐induced muscle atrophy models was analysed by western blots (*n* = 4). Multiple group comparisons were conducted using one‐way or two‐way ANOVA, followed by Dunnett’s T3 or Bonferroni post hoc tests, depending on the homogeneity of variance assessed. The statistical results were represented by Mean ± SD. ***p < 0.001.
**Figure S7.** Inhibition of EIF4A3 expression does not affect the myogenic differentiation and fast‐ and slow‐type isoforms during dexamethasone treatment. (A) The myotube fusion index of C2C12 myotubes transfected with sh‐EIF4A3#1 and sh‐EIF4A3#2 lentivirus in a dexamethasone (Dex)‐induced myotube (*n* = 4 per group). (B) Analysis of myogenic differentiation‐associated genes and fast‐ and slow‐type isoform‐associated genes in C2C12 myotubes transfected with sh‐EIF4A3#1 and sh‐EIF4A3#2 lentivirus in a dexamethasone (Dex)‐induced myotube (*n* = 4). (C and D) Western blot analysis of MyoD, fast or slow myosin heavy chain (MyHC) protein expression levels in C2C12 myotubes transfected with sh‐EIF4A3#1 and sh‐EIF4A3#2 lentivirus in a dexamethasone (Dex)‐induced myotube (*n* = 4). followed by Dunnett’s T3 or Bonferroni post hoc tests, depending on the homogeneity of variance assessed. The statistical results were represented by Mean ± SD.
**Figure S8.** Inhibition of EIF4A3 expression prevents muscle atrophy caused by denervation in vivo. (A) Experimental design process and schematic diagram of virus injection dose. (B) The expression level of *Eif4a3* in gastrocnemius of AAV8‐sh‐EIF4A3 and AAV8‐scramble mice was detected by RT‐qPCR (*n* = 10). (C) The expression level of *Fbxo32* and *Trim63* in gastrocnemius of AAV8‐sh‐EIF4A3 and AAV8‐scramble mice was detected by RT‐qPCR (*n* = 10). (D) Western blot analysis of protein synthesis by anti‐Puromycin in muscle of mice injected with AAV8‐sh‐EIF4A3 and AAV8‐scramble (*n* = 6). (E) The types of gastrocnemius fibres in muscle of mice injected with AAV8‐sh‐EIF4A3 and AAV8‐scramble were detected by immunofluorescence staining (*n* = 7–11). (F) Western blot analysis of fast or slow myosin heavy chain (MyHC) protein expression levels in muscle of mice injection with AAV8‐sh‐EIF4A3 and AAV8‐scramble during denervation (*n* = 6). Multiple group comparisons were conducted using one‐way or two‐way ANOVA, followed by Dunnett’s T3 or Bonferroni post hoc tests, depending on the homogeneity of variance assessed. The statistical results were represented by Mean ± SD. ***p < 0.001.
**Figure S9.** Inhibition of EIF4A3 expression prevents muscle atrophy caused by immobilization in vivo. (A) Experimental design process and schematic diagram of virus injection dose. (B) The expression level of *Eif4a3* in the gastrocnemius of AAV8‐sh‐EIF4A3 and AAV8‐scramble mice was detected by RT‐qPCR (*n* = 10). (C) Western blot analysis of EIF4A3 protein expression in gastrocnemius of mice injected with AAV8‐sh‐EIF4A3 and AAV8‐scramble (*n* = 6). (D) Statistical analysis of grip strength of right hind limb muscles in mice injected with AAV8‐sh‐EIF4A3 and AAV8‐scramble (*n* = 10). (E) Running distance of mice injected with AAV8‐sh‐EIF4A3 and AAV8‐scramble (*n* = 10). (F) Representative images of morphology and body weight (G/BW) of gastrocnemius in mice injected with AAV8‐sh‐EIF4A3 and AAV8‐scramble (*n* = 10, scale: 1 cm). (G) Representative images and statistical analysis of muscle fibre cross‐sectional area in mice injected with AAV8‐sh‐EIF4A3 and AAV8‐scramble (*n* = 6,8,6,6; Bar: 50 μm). (H) Expression levels of MuRF‐1 and Atrogin‐1 in gastrocnemius of mice injected with AAV8‐sh‐EIF4A3 and AAV8‐scramble were detected by western blot (*n* = 6). (I) Expression levels of *Fbxo32* and *Trim63* genes in gastrocnemius of mice injected with AAV8‐sh‐EIF4A3 and AAV8‐scramble were detected by RT‐qPCR (*n* = 10). (J) Western blot analysis of protein synthesis by anti‐Puromycin in gastrocnemius of mice injected with AAV8‐sh‐EIF4A3 and AAV8‐scramble (*n* = 6). Multiple group comparisons were conducted using one‐way or two‐way ANOVA, followed by Dunnett’s T3 or Bonferroni post hoc tests, depending on the homogeneity of variance assessed. The statistical results were represented by Mean ± SD. **p < 0.01, ***p < 0.001.
**Figure S10.** Inhibition of EIF4A3 expression prevents angiotensin II‐induced muscle atrophy in vivo. (A) Experimental design process and schematic diagram of virus injection dose. (B) The expression level of *Eif4a3* gene in the gastrocnemius of AAV8‐sh‐EIF4A3 and AAV8‐scramble mice was detected by RT‐qPCR (*n* = 10, 8, 6, 8). (C) Western blot analysis of the expression of EIF4A3 protein in gastritis muscle of mice injected with AAV8‐sh‐EIF4A3 and AAV8‐scramble (*n* = 6). (D) Statistical analysis of grip strength in right hind limb muscles of mice injected with AAV8‐sh‐EIF4A3 and AAV8‐scramble (*n* = 10, 8, 6, 8). (E) Running distance of mice injected with AAV8‐sh‐EIF4A3 and AAV8‐scramble (*n* = 10, 8, 6, 8). (F) Representative images of morphology and the ratio of skeletal muscle (Gastrocnemius muscle, G; Tibialis anterior muscle, TA) weight and tibial length (TL) of gastrocnemius muscle in mice injected with AAV8‐sh‐EIF4A3 and AAV8‐scramble (*n* = 10, 8, 6, 8, scale: 1 cm). (G) Representative images and statistical analysis of muscle fibre cross‐sectional area and TUNEL staining in mice injected with AAV8‐sh‐EIF4A3 and AAV8‐scramble (*n* = 10, 8, 6, 8; Bar: 50 μm). (H) Expression levels of MuRF‐1 and Atrogin‐1 protein in gastrocnemius of mice injected with AAV8‐sh‐EIF4A3 and AAV8‐scramble were detected by western blot (*n* = 10, 8, 6, 8). (I) The expression level of *Fbxo32* and *Trim63* gene in the gastrocnemius of AAV8‐sh‐EIF4A3 and AAV8‐scramble mice was detected by RT‐qPCR (*n* = 10, 8, 6, 8). (J) Western blot analysis of protein synthesis by anti‐Puromycin in gastrocnemius of mice injected with AAV8‐sh‐EIF4A3 and AAV8‐scramble (*n* = 6). Multiple group comparisons were conducted using one‐way or two‐way ANOVA, followed by Dunnett’s T3 or Bonferroni post hoc tests, depending on the homogeneity of variance assessed. The statistical results were represented by Mean ± SD. **p < 0.01, ***p < 0.001.
**Figure S11.** Filter out NEDD9 mRNA to be the downstream of EIF4A3. (A) Volcanoplots showing inclusion level differences in various ASEs between EIF4A3 overexpression and controls. (B) Bar graphs showing the enriched terms across splicing‐altered genes. (C) Venn diagrams showing the overlap between dysregulated genes and genes with altered splicing in EIF4A3 overexpression. (D) Venn diagrams diagram for the overlap between dysregulated genes and RNAs binding with EIF4A3. And Venn diagrams showing the overlap between dysregulated genes in EIF4A3 overexpression and EIF4A3 binding mRNA. (E) Differential genes were screened by C2C12 myotubes treated with EIF4A3‐OE and sh‐EIF4A3 lentivirus (*n* = 4). Further screening was performed by (F) cell muscle atrophy models (*n* = 4) and (G) mice muscle atrophy models (*n* = 4). (H) RT‐qPCR analysis of *Nedd9* expression levels in gastrocnemius muscle tissues of mice muscle atrophy models in female mice (*n* = 4 per group). (I) Subcellular distribution of *Nedd9* mRNA and EIF4A3 in baseline and EIF4A3 OE treated myotube. The comparison between two groups was performed using Student’s *t*‐test. *p < 0.05, **p < 0.01, ***p < 0.001.
**Figure S12.** EIF4A3 facilitates muscle atrophy via binding the NEDD9 mRNA and promoting RNA degradation. (A) Western Blot was used to detect the expression level of NEDD9 in C2C12 myotubes transfected with sh‐NEDD9 lentivirus (*n* = 6). (B) Representative images and statistical analysis of C2C12 myotubes transfected with sh‐NEDD9 and control lentivirus (*n* = 4), scale: 100 μm. (C) Expression levels of *Fbxo32* and *Trim63* genes in C2C12 myotube transfected with sh‐NEDD9 and controls lentivirus were evaluated by RT‐qPCR (*n* = 6). (D) Expression levels of MuRF‐1 and Atrogin‐1 proteins in C2C12 myotube transfected with sh‐NEDD9 and controls lentivirus were evaluated by western blot (*n* = 6). (E)Western Blot was used to detect the expression level of NEDD9 in C2C12 myotubes transfected with NEDD9‐OE lentivirus (*n* = 6). (F) The changes of myotubes’ diameter in C2C12 myotubes treated with EIF4A3‐OE and NEDD9‐OE lentivirus were detected by immunofluorescence (*n* = 4). (G) The expression levels of *Fbxo32* and *Trim63* genes in C2C12 myotubes treated with EIF4A3‐OE and NEDD9‐OE lentivirus were detected by RT‐qPCR (*n* = 6). (H) The expression levels of MuRF‐1 and Atrogin‐1 proteins in C2C12 myotubes treated with EIF4A3‐OE and NEDD9‐OE lentivirus were detected by western blot (*n* = 6). The comparison between two groups was performed using Student’s *t*‐test (A‐E). Multiple group comparisons were conducted using one‐way or two‐way ANOVA, followed by Dunnett’s T3 or Bonferroni post hoc tests, depending on the homogeneity of variance assessed (F‐H). The statistical results were represented by Mean ± SD. ***p < 0.001.
**Figure S13.** Inactivation of NEDD9‐FAK pathway in muscle atrophy model and muscle aging. Western blot analysis of FAK pathway expression in (A) muscle atrophy model (*n* = 3 in male, *n* = 4 in female) and C2C12 cell atrophy model (B) (*n* = 3). Western blot analysis of FAK pathway protein expression in (C) aged human muscle (*n* = 4) and (D) aged mouse gastrocnemius tissue (*n* = 6 in male, *n* = 6 in female). RT‐qPCR analysis of NEDD9 mRNA expression in (E) aged human muscle (*n* = 5) and (F) aged mouse gastrocnemius tissue (*n* = 6 in male, *n* = 6 in female). The comparison between two groups was performed using Student’s *t*‐test. The statistical results were represented by Mean ± SD. *p < 0.05, **p < 0.01, ***p < 0.001.
**Figure S14.** SAHA stimulation of NEDD9‐FAK signalling pathway can alleviate muscle atrophy induced by EIF4A3 overexpression in vitro. (A) Western Blot analysis of the expression levels of NEDD9 and FAK pathway proteins in C2C12 myotubes treated with EIF4A3‐OE lentivirus and SAHA (*n* = 6). (B) The change of myotubes’ diameter of C2C12 myotube after treatment of EIF4A3‐OE lentivirus and SAHA was detected by immunofluorescence (*n* = 4). (C) The expression levels of MuRF‐1 and Atrogin‐1 proteins in C2C12 myotubes after transfection with EIF4A3‐OE and SAHA were detected by western blot (*n* = 6). (D) The expression levels of *Fbxo32* and *Trim63* genes in C2C12 myotubes after transfection with EIF4A3‐OE and SAHA were detected by RT‐qPCR (*n* = 6). Multiple group comparisons were conducted using one‐way or two‐way ANOVA, followed by Dunnett’s T3 or Bonferroni post hoc tests, depending on the homogeneity of variance assessed. The statistical results were represented by Mean ± SD. **p < 0.01, ***p < 0.001.
**Figure S15.** SAHA stimulation of NEDD9‐FAK signalling pathway can alleviate muscle atrophy induced by EIF4A3 overexpression in vivo. (A) Experimental design process and schematic diagram of virus injection dose. (B) Expression levels of *Fbxo32* and *Trim63* genes in gastrocnemius of mice injected with AAV8‐EIF4A3‐OE and SAHA were detected by RT‐qPCR (*n* = 8). (C)Western blot analysis of Puromycin protein expression in gastrocnemius of mice injected with AAV8‐EIF4A3‐OE and SAHA (*n* = 6). (D) The types of gastrocnemius fibres in muscle of mice injected with AAV8‐EIF4A3‐OE and SAHA were detected by immunofluorescence staining (*n* = 12–14). (E) Western blot analysis of fast or slow myosin heavy chain (MyHC) protein expression levels in muscle of mice injected with AAV8‐EIF4A3‐OE and SAHA (*n* = 6). Multiple group comparisons were conducted using one‐way or two‐way ANOVA, followed by Dunnett’s T3 or Bonferroni post hoc tests, depending on the homogeneity of variance assessed. The statistical results were represented by Mean ± SD. **p < 0.01, ***p < 0.001.
**Table S1.** Primers used in this study.

## References

[1] A. J. Cruz‐ Jentoft and A. A. Sayer , “Sarcopenia,” Lancet 393 (2019): 2636–2646. 31171417 10.1016/S0140-6736(19)31138-9

[2] W. Q. Xie , M. He , D. J. Yu , et al., “Mouse Models of Sarcopenia: Classification and Evaluation,” Journal of Cachexia, Sarcopenia and Muscle 12 (2021): 538–554. 33951340 10.1002/jcsm.12709 PMC8200444

[3] L. M. Baehr , D. C. Hughes , D. S. Waddell , and S. C. Bodine , “SnapShot: Skeletal Muscle Atrophy,” Cell 185 (2022): 1618–e1. 35487192 10.1016/j.cell.2022.03.028

[4] R. Sartori , V. Romanello , and M. Sandri , “Mechanisms of Muscle Atrophy and Hypertrophy: Implications in Health and Disease,” Nature Communications 12 (2021): 330. 10.1038/s41467-020-20123-1 PMC7803748 33436614

[5] H. Gallagher , P. W. Hendrickse , M. G. Pereira , and T. S. Bowen , “Skeletal Muscle Atrophy, Regeneration, and Dysfunction in Heart Failure: Impact of Exercise Training,” Journal of Sport and Health Science 12 (2023): 557–567. 37040849 10.1016/j.jshs.2023.04.001 PMC10466197

[6] Y. Shen , Q. Shi , K. Nong , et al., “Exercise for Sarcopenia in Older People: A Systematic Review and Network Meta‐Analysis,” Journal of Cachexia, Sarcopenia and Muscle 14 (2023): 1199–1211. 37057640 10.1002/jcsm.13225 PMC10235889

[7] M. Guo , F. Shen , X. Guo , et al., “BMAL1/PGC1alpha4‐FNDC5/Irisin Axis Impacts Distinct Outcomes of Time‐Of‐Day Resistance Exercise,” Journal of Sport and Health Science 14 (2024): 100968. 39187065 10.1016/j.jshs.2024.100968 PMC11863284

[8] V. Boehm and N. H. Gehring , “Exon Junction Complexes: Supervising the Gene Expression Assembly Line,” Trends in Genetics 32 (2016): 724–735. 27667727 10.1016/j.tig.2016.09.003

[9] L. A. Woodward , J. W. Mabin , P. Gangras , and G. Singh , “The Exon Junction Complex: A Lifelong Guardian of mRNA Fate,” Wiley Interdisciplinary Reviews: RNA 8 (2017): e1411. 10.1002/wrna.1411 28008720

[10] H. Le Hir , J. Sauliere , and Z. Wang , “The Exon Junction Complex as a Node of Post‐Transcriptional Networks,” Nature Reviews Molecular Cell Biology 17 (2016): 41–54. 26670016 10.1038/nrm.2015.7

[11] L. Ballut , B. Marchadier , A. Baguet , C. Tomasetto , B. Seraphin , and H. Le Hir , “The Exon Junction Core Complex Is Locked Onto RNA by Inhibition of eIF4AIII ATPase Activity,” Nature Structural & Molecular Biology 12 (2005): 861–869. 10.1038/nsmb990 16170325

[12] P. Linder and E. Jankowsky , “From Unwinding to Clamping—The DEAD Box RNA Helicase Family,” Nature Reviews Molecular Cell Biology 12 (2011): 505–516. 21779027 10.1038/nrm3154

[13] F. Bono , J. Ebert , E. Lorentzen , and E. Conti , “The Crystal Structure of the Exon Junction Complex Reveals How It Maintains a Stable Grip on mRNA,” Cell 126 (2006): 713–725. 16923391 10.1016/j.cell.2006.08.006

[14] S. Miliara , E. Cozzi , X. Zhong , et al., “The Exon‐Junction Complex Helicase eIF4A3 Holds Therapeutic Potential in Acute Myeloid Leukemia,” Leukemia 38 (2024): 663–666. 38036629 10.1038/s41375-023-02098-2 PMC10912025

[15] R. Blazquez‐Encinas , E. Alors‐Perez , M. T. Moreno‐Montilla , et al., “The Exon Junction Complex Component EIF4A3 Plays a Splicing‐Linked Oncogenic Role in Pancreatic Ductal Adenocarcinoma,” Cancer Gene Therapy 31 (2024): 1646–1657. 39232176 10.1038/s41417-024-00814-3 PMC11567885

[16] D. Li , J. Yang , V. Malik , et al., “An RNAi Screen of RNA Helicases Identifies eIF4A3 as a Regulator of Embryonic Stem Cell Identity,” Nucleic Acids Research 50 (2022): 12462–12479. 36416264 10.1093/nar/gkac1084 PMC9757061

[17] X. Zhu , T. Yang , Y. Zheng , et al., “EIF4A3‐Induced Circular RNA CircDdb1 Promotes Muscle Atrophy Through Encoding a Novel Protein CircDdb1‐867aa,” Advanced Science (Weinheim) 11 (2024): e2406986. 10.1002/advs.202406986 PMC11615752 39412095

[18] J. Li , M. C. Chan , Y. Yu , et al., “miR‐29b Contributes to Multiple Types of Muscle Atrophy,” Nature Communications 8 (2017): 15201. 10.1038/ncomms15201 PMC5458521 28541289

[19] Q. Liu , L. Chen , X. Liang , et al., “Exercise Attenuates AngiotensinII‐Induced Muscle Atrophy by Targeting PPARgamma/miR‐29b,” Journal of Sport and Health Science 11 (2022): 696–707. 34116237 10.1016/j.jshs.2021.06.002 PMC9729927

[20] J. Li , T. Yang , Z. Sha , et al., “Angiotensin II‐Induced Muscle Atrophy via PPARgamma Suppression is Mediated by miR‐29b,” Molecular Therapy—Nucleic Acids 23 (2021): 743–756. 33614226 10.1016/j.omtn.2020.12.015 PMC7868689

[21] J. Li , T. Yang , H. Tang , et al., “Inhibition of lncRNA MAAT Controls Multiple Types of Muscle Atrophy by cis‐ and Trans‐Regulatory Actions,” Molecular Therapy 29 (2021): 1102–1119. 33279721 10.1016/j.ymthe.2020.12.002 PMC7934634

[22] H. Lin , H. Peng , Y. Sun , et al., “Reprogramming of cis‐Regulatory Networks During Skeletal Muscle Atrophy in Male Mice,” Nature Communications 14 (2023): 6581. 10.1038/s41467-023-42313-3 PMC10584982 37853001

[23] V. R. Kedlian , Y. Wang , T. Liu , et al., “Human Skeletal Muscle Aging Atlas,” Nature Aging 4 (2024): 727–744. 38622407 10.1038/s43587-024-00613-3 PMC11108788

[24] E. Szostak and F. Gebauer , “Translational Control by 3′‐UTR‐Binding Proteins,” Briefings in Functional Genomics 12 (2013): 58–65. 23196851 10.1093/bfgp/els056 PMC3548161

[25] G. Tushev , C. Glock , M. Heumuller , A. Biever , M. Jovanovic , and E. M. Schuman , “Alternative 3′ Utrs Modify the Localization, Regulatory Potential, Stability, and Plasticity of mrnas in Neuronal Compartments,” Neuron 98 (2018): 495, e6–511. 29656876 10.1016/j.neuron.2018.03.030

[26] M. K. Singh , D. Dadke , E. Nicolas , et al., “A Novel Cas Family Member, HEPL, Regulates FAK and Cell Spreading,” Molecular Biology of the Cell 19 (2008): 1627–1636. 18256281 10.1091/mbc.E07-09-0953 PMC2291417

[27] N. Sima , X. Cheng , F. Ye , D. Ma , X. Xie , and W. Lu , “The Overexpression of Scaffolding Protein NEDD9 Promotes Migration and Invasion in Cervical Cancer Via Tyrosine Phosphorylated FAK and SRC,” PLoS ONE 8 (2013): e74594. 24058594 10.1371/journal.pone.0074594 PMC3776827

[28] Z. Hu , F. Wei , Y. Su , et al., “Histone Deacetylase Inhibitors Promote Breast Cancer Metastasis by Elevating NEDD9 Expression,” Signal Transduction and Targeted Therapy 8 (2023): 11. 36604412 10.1038/s41392-022-01221-6 PMC9816171

[29] M. Yang , H. Xiang , and G. Luo , “Targeting Focal Adhesion Kinase (FAK) for Cancer Therapy: FAK Inhibitors, FAK‐Based Dual‐Target Inhibitors and PROTAC Degraders,” Biochemical Pharmacology 224 (2024): 116246. 38685282 10.1016/j.bcp.2024.116246

[30] R. Paul , M. Luo , X. Mo , J. Lu , S. K. Yeo , and J. L. Guan , “FAK Activates AKT‐mTOR Signaling to Promote the Growth and Progression of MMTV‐Wnt1‐Driven Basal‐Like Mammary Tumors,” Breast Cancer Research 22 (2020): 59. 32493400 10.1186/s13058-020-01298-3 PMC7268629

[31] X. Jiang , S. Guo , S. Wang , et al., “EIF4A3‐Induced circARHGAP29 Promotes Aerobic Glycolysis in Docetaxel‐Resistant Prostate Cancer Through IGF2BP2/c‐Myc/LDHA Signaling,” Cancer Research 82 (2022): 831–845. 34965937 10.1158/0008-5472.CAN-21-2988

[32] D. Da Cunha , J. Miro , C. Van Goethem , et al., “The Exon Junction Complex Is Required for DMD Gene Splicing Fidelity and Myogenic Differentiation,” Cellular and Molecular Life Sciences 81 (2024): 150. 38512499 10.1007/s00018-024-05188-1 PMC10957711

[33] M. Iwatani‐Yoshihara , M. Ito , Y. Ishibashi , et al., “Discovery and Characterization of a Eukaryotic Initiation Factor 4A‐3‐Selective Inhibitor That Suppresses Nonsense‐Mediated mRNA Decay,” ACS Chemical Biology 12 (2017): 1760–1768. 28440616 10.1021/acschembio.7b00041

[34] R. Cencic , S. K. Naineni , L. Pugsley , P. Senechal , A. Sahni , and J. Pelletier , “CRISPR‐Based Screen Links an Inhibitor of Nonsense‐Mediated Decay to eIF4A3 Target Engagement,” ACS Chemical Biology 15 (2020): 1621–1629. 32401488 10.1021/acschembio.0c00253

[35] F. C. Alsina , B. M. Lupan , L. J. Lin , et al., “The RNA‐Binding Protein EIF4A3 Promotes Axon Development by Direct Control of the Cytoskeleton,” Cell Reports 43 (2024): 114666. 39182224 10.1016/j.celrep.2024.114666 PMC11488691

[36] D. C. Kanellis , J. A. Espinoza , A. Zisi , et al., “The Exon‐Junction Complex Helicase eIF4A3 Controls Cell Fate via Coordinated Regulation of Ribosome Biogenesis and Translational Output,” Science Advances 7 (2021): eabf7561. 34348895 10.1126/sciadv.abf7561 PMC8336962

[37] W. Liu and G. Luo , “NEDD9 Is Transcriptionally Regulated by HDAC4 and Promotes Breast Cancer Metastasis and Macrophage M2 Polarization via the FAK/NF‐kappaB Signaling Pathway,” Neoplasia 57 (2024): 101059. 39326322 10.1016/j.neo.2024.101059 PMC11470473

[38] M. Fluck , R. Li , P. Valdivieso , et al., “Early Changes in Costameric and Mitochondrial Protein Expression With Unloading Are Muscle Specific,” BioMed Research International 2014 (2014): 519310. 25313365 10.1155/2014/519310 PMC4182083

[39] S. E. Gordon , M. Fluck , and F. W. Booth , “Skeletal Muscle Focal Adhesion Kinase, Paxillin, and Serum Response Factor Are Loading Dependent,” Journal of Applied Psychology (1985) 90 (2001): 1174–1183 discussion 65. 10.1152/jappl.2001.90.3.1174 11181634

[40] S. Klossner , A. C. Durieux , D. Freyssenet , and M. Flueck , “Mechano‐Transduction to Muscle Protein Synthesis Is Modulated by FAK,” European Journal of Applied Physiology 106 (2009): 389–398. 19294408 10.1007/s00421-009-1032-7

[41] R. Li , M. V. Narici , R. M. Erskine , et al., “Costamere Remodeling With Muscle Loading and Unloading in Healthy Young Men,” Journal of Anatomy 223 (2013): 525–536. 24010829 10.1111/joa.12101 PMC3916893

[42] Z. A. Graham , P. M. Gallagher , and C. P. Cardozo , “Focal Adhesion Kinase and Its Role in Skeletal Muscle,” Journal of Muscle Research and Cell Motility 36 (2015): 305–315. 26142360 10.1007/s10974-015-9415-3 PMC4659753

[43] S. von Haehling , A. J. S. Coats , and S. D. Anker , “Ethical Guidelines for Publishing in the Journal of Cachexia, Sarcopenia and Muscle: Update 2021,” Journal of Cachexia, Sarcopenia and Muscle 12 (2021): 2259–2261. 34904399 10.1002/jcsm.12899 PMC8718061

