# Supplementary material for: EIF4A3 Promotes Muscle Atrophy and Aging by Inhibiting the FAK Pathway Through NEDD9 mRNA Destabilization
Source: J Cachexia Sarcopenia Muscle. 2025 Jul 10;16(4):e70010. doi: 10.1002/jcsm.70010 (PMC12246388; doi:10.1002/jcsm.70010)
Supplement: Supplementary file 1 — Figure S1. Expression of EIF4A3 in atrophic muscle in female mice. Western blot analysis of EIF4A3 expression levels in gastrocnemius muscle tissues of denervation (Den)‐ (A), immobilization (Imo)‐ (B) and Angiotensin II (Ang II)‐ (C) induced muscle atrophy models in female mice (n = 4 per group). The comparison between two groups was performed using Student’s t‐test. The statistical results were represented by Mean ± SD. **p < 0.01, ***p < 0.001. Figure S2. EIF4A3 overexpression does not affect the myogenic differentiation and fast‐ and slow‐type isoforms in mice myotube cells. (A) The myotube fusion index of C2C12 myotubes transfected with EIF4A3‐OE for 2 days (n = 4 per group). (B) Analysis of myogenic differentiation‐associated genes and fast‐ and slow‐type isoform‐associated genes in C2C12 myotubes transfected with EIF4A3‐OE and FUGW lentivirus were evaluated by RT‐qPCR (n = 6). (C and D) Western blot analysis of MyoD, fast or slow myosin heavy chain (MyHC) protein expression levels in C2C12 myotubes transfected with EIF4A3‐OE and control lentivirus (n = 6). The comparison between two groups was performed using Student’s t‐test. The statistical results were represented by Mean ± SD. Figure S3. EIF4A3 promotes muscle atrophy and muscle aging in vitro. (A) Expression levels of Eif4a3 in C2C12 myotubes transfected with EIF4A3 overexpression (EIF4A3‐OE) and controls (FUGW) lentivirus were evaluated by RT‐qPCR (n = 6). (B) Expression levels of Fbxo32 and Trim63 genes in C2C12 myotube transfected with EIF4A3‐OE and controls lentivirus were evaluated by RT‐qPCR (n = 6). (C) Western blot analysis of ubiquitin–protein expression in C2C12 myotubes transfected with EIF4A3‐OE and control lentivirus (n = 6). (D) Western blot analysis of P62 and LC3 protein expression levels in C2C12 myotubes transfected with EIF4A3‐OE and control lentivirus (n = 6). (E) Western blot analysis of caspase 3 and Bax/Bcl2 protein expression levels in C2C12 myotubes transfected with EIF4A3‐OE and [file JCSM-16-e70010-s001.docx]

**1. Methods**

**1.1 Antibodies and Reagents**

The antibodies used were as follows: EIF4A3 antibody (17504-1-AP, Proteintech, China), FAK antibody (12636-1-AP, Proteintech, China), Phospho-FAK(Y397) (ET1610-34, Huabio, China), NEDD9 Rabbit pAB (A2521, ABclonal, China), Rabbit (DA1E) mAb IgG XP Isotype Control (3900S, CST, USA). PI3K(p85α) (A12305, ABclonal, China), Phospho-PI3K(p85α) (AP0854, ABclonal, China), AKT antibody (10176-2-AP, Proteintech, China), Phospho-AKT(Ser473) (66444-1-Ig, Proteintech, China), mTOR antibody (2972s, CST, USA), Phospho-mTOR (2971s, CST, USA), MyoD (18943-1-AP, Proteintech, China), MyHC(fast) (A27146, ABclonal, China), MyHC(slow) (BA-D5, DSHB, USA), TRIM63 (55456-1-AP, Proteintech, China), FBXO32 (67172-1-1g, Proteintech, China). Antibodies were diluted according to the manufacturer's instructions. The following reagents were used: SAHA (S1047, Selleck, USA), Protein A+G Beads (P2108, Beyotime, China).

**1.2 Single cell RNA sequencing (scRNA seq) analysis**

Single-cell RNA sequencing data from muscle tissues of muscle atrophy model were obtained from the Gene Expression Omnibus (GEO) database (GSE183802) as well as ArrayExpress (E-MTAB-13874)[1, 2]. Cells expressing fewer than 200 or more than 10,000 genes were excluded to remove non-cellular debris and potential cell aggregates. The data were log-normalized, and highly variable genes were identified using the Find Variable Features function. Subsequent analyses, including data scaling and principal component analysis (PCA), were conducted using the Scale Data and Run PCA functions, respectively. Cell clustering was performed with the Find Neighbors and Find Clusters functions, and uniform manifold approximation and projection (UMAP) was used for cluster visualization, implemented in the Seurat R package as per the official vignettes (https://satijalab.org/seurat/articles/get_started.html). Cluster annotation was performed based on known cell markers.

**1.3 Cell Culture and Transfection**

C2C12 myoblasts and HEK293T cells were obtained from ATCC in a compound containing 10% heat-inactivated fetal bovine serum (ExCell Bio, China) and 1% penicillin-streptomycin (KeyGEN, China). The cells were cultured at 37°C with 5% CO_2_. C2C12 myoblasts are passed before reaching 80% confluence to maintain their undifferentiated state. The differentiation of C2C12 myoblasts was initiated using a differentiation medium, which consisted of 2% horse serum and 1% penicillin-streptomycin mixed in Dulbecco's Modified Eagle Medium (DMEM), when the cells reached a confluence of 70%. After 4 days of differentiation, multinucleated muscle tubes were formed, and all C2C12 myotubes experiments were performed by using fully differentiated cells.

To assess the function of EIF4A3 or NEDD9 in muscle atrophy *in vitro*, after differentiation for 4 days to obtain mature myotube, then myotubes transfection was performed with lentivirus at MOI 100 for 24h. After that differentiation medium was mixed with 50μM Dex (or 100 ng/ml TNF-α or 500nM AngII) and incubated for 24 hours (see Figure 4B, S6, S7). If myotube atrophy didn’t induced by 50μM Dex (or 100 ng/ml TNF-α or 500nM AngII), after differentiation for 4 days to obtain mature myotube, then myotubes transfection was performed with lentivirus at MOI 100 for 48h. (see Figure 2B, S2, S12B, S12F, S14).

Human skeletal muscle myoblasts were obtained from ZQXZbio (PRI-H-00152, shanghai ZhongQiao Xin Zhou Biotechnology co.,Ltd, China). The cells were cultured in growth medium with 10% heat-inactivated fetal bovine serum (ExCell Bio, China) and 1% penicillin-streptomycin (KeyGEN, China). To obtain human myotube cell, differentiation medium (DMEM medium containing 2% horse serum, 1% penicillin-streptomycin and 10 μg/mL insulin) were used to treat for 5 days. After that, the mature myotube human myotube transfected with lentivirus at MOI 100 for 48h. and the treated cells were collected for further analyze (see Figure 3, Figure S5).

**1.4 Western Blotting**

Samples of gastrocnemius and treated C2C12 myotubes were lysed by lysis buffer containing protease and phosphatase inhibitors (KeyGEN, China), and the proteins were quantified using BCA protein assays (TaKaRa, Japan). SDS-PAGE electrophoresis was performed of the same amount of protein and transferred to PVDF membrane. After blocking with 5% skim milk, the membrane was cleaned with PBS containing 0.05% Tween-20 and incubated with primary antibody at 4°C overnight. Then, the membrane was cleaned and incubated in 5% skim milk containing secondary antibodies. The protein expression was detected using the ECL chemiluminescence kit (Tanon, China) and the chemiluminescence imaging of the membrane was detected by the BioRad luminescence imaging system. The protein bands were quantified using Image J software.

**1.5 Immunofluorescence Staining**

Samples were prepared for immunostaining by fixing frozen sections or cells in 4% paraformaldehyde (PFA) for 20 minutes at room temperature. The samples were then permeabilized with 0.5% Triton X-100 for 15 minutes, blocked with 5% BSA for 2 hours, and incubated overnight at 4°C with primary antibodies. The following day, the samples were incubated with secondary antibodies for 1 hour at room temperature, protected from light. The nuclei were incubated with Hoechst dye (KeyGEN, China) at room temperature. Fluorescence images were captured using an inverted fluorescence microscope (Leica, Germany; Olympus, Japan) and myofiber diameter was measured with Image J software. In muscle fibre-type determination, primary antibodies used as followed: MHCI (1:50, BA-F8, DSHB), MHCIIa (1:50, SC-71, DSHB) and MHCIIb (1:50, BF-F3, DSHB). The secondary antibodies used as follows: Alexa Fluor 350 anti-mouse IgG2b (A-21140, Thermo Fisher), Alexa Fluor 488 anti-mouse IgG1 (A-21121, Thermo Fisher) and Alexa Fluor 555 anti-mouse IgM (A-21426, Thermo Fisher).

**1.6 Actinomycin D test (ActD)**

After the cells were treated, 10μg/ml actinomycin D (Sigma, Germany) was administered at 0h, 2h, 4h, 6h, 8h, 10h time points, and samples were collected in batches for real-time fluorescence quantitative PCR detection and analysis.

**1.7 RNA Isolation and Real-Time Quantitative PCR (RT-qPCR)**

Total RNA was isolated from tissues and C2C12 myotubes and muscle tissues by using RNAiso Plus (Takara, Japan). The cDNA was synthesized using RevertAid First Strand cDNA Synthesis Kit (Thermo, USA). Real-time quantitative PCR was performed using ChamQ Universal SYBR qPCR Master Mix (Vazyme, China). The expression level of the target gene was normalized to 18s, and the multiples were calculated using 2^-ΔΔCt^ method. The primers were listed in Table S1.

**1.8 RNA immunoprecipitation (RIP)**

RIP buffer (Including KCl, Tris-HCl, EDTA, NP-40 and ddH2O, and DTT, RNAse OUT, Protease inhibitor) was used to lyse the cells, which were rotated at 4℃ for 20min and ultrasonically broken for 5min to fully lyse. The supernatant was centrifuged and incubated with the protein G beads cleaned with RIP buffer at 4℃ for 1h to remove specific adsorption. The upper supernatant was divided into IgG group and EIF4A3 antibody (17504-1-AP, Proteintech, China) group, incubated overnight, and beads were added for another 2h the next day. After cleaning, immunoprecipitation sample were used for RNA extraction and further detection.

**1.9 RNA Electrophoretic Mobility Shift Assay (REMSA)**

REMSA was performed using the LightShift Chemiluminescent RNA EMSA Kit (Thermo, USA). The 10μl reaction mixture contained 2μM probe, REMSA Binding Buffer, and 2μg EIF4A3 protein (EIF4A3: Ag11130, Proteintech, USA). After incubation, samples were electrophoresed on a 5% TBE-Urea gel, transferred to nylon film, and cross-linked. The film was then blocked, incubated with Streptavidin-HRP, and detected using the ECL chemiluminescence kit (Thermo Fisher, USA) and BioRad luminescence imaging system.

**1.10 RNA Pulldown**

C2C12 myotube cells were collected from a petri dish and lysis was performed by adding Cell Lysis Buffer (1 M Tris-HCl pH 7.0, 0.5 M EDTA, 20% SDS, 1.25 M DTT, 40 U/μl RNAseOut, Protease inhibitor). Samples were ground and centrifuged, then the supernatant was collected. A portion was set aside as the Input group, while the rest was incubated with a biotin-labeled probe (and Random probe) and Hybridization buffer (1 M Tris-HCl pH 7.0, 0.5 M EDTA, 20% SDS, 5 M NaCl, Formamide, 40 U/μl RNAseOut, Protease inhibitor) at room temperature on the rotator overnight. Pierce Streptavidin Magnetic Beads (Thermo, USA) were added and incubated at room temperature on a rotary apparatus for 3 hours. The magnetic beads were stored and dissolve. Then extract RNA and protein for further detection. The probes used as followed: Probe-1: Biotin-aaaTATATTCTCCAAAAAAAAAAAAAAAAAAAAAAAAAGGA. Probe-2: Biotin-aaaATATCACCAGGAAGATTAAACATCACAACTATAAGAGGC

**1.11 Muscle function testing**

*Grip-strength measurement:* Mice were placed on a digital grip-strength meter (YLS-13A, Yiyan Technology Co. Ltd, China) and allowed to adapt and hold onto the grid. The average grip strength of the mice's limbs without dropping was measured and recorded by gently pulling their tails.

*Treadmill exercise:* Mice were acclimated to the treadmill and then subjected to a graded exercise protocol[3]. The treadmill was initiated at a low speed, which was incrementally increased to the target velocity. Exercise duration was recorded until the mice exhibited visible signs of exhaustion.

*Contractility measurement:* The right extensor digitorum longus (EDL) muscle of the hind limb from virus-injected mice was dissected and attached to surgical wires at both ends. The muscle was then suspended from a force transducer (Aurora Scientific, Canada) to measure isometric contractile force in response to 1A electrical stimulation.

**Reference**

1. Lin H, Peng H, Sun Y, Si M, Wu J, Wang Y, et al. Reprogramming of cis-regulatory networks during skeletal muscle atrophy in male mice. Nat Commun. 2023;14:6581.

2. Kedlian VR, Wang Y, Liu T, Chen X, Bolt L, Tudor C, et al. Human skeletal muscle aging atlas. Nat Aging. 2024;4:727-44.

3. Zhu X, Yang T, Zheng Y, Nie Q, Chen J, Li Q, et al. EIF4A3-Induced Circular RNA CircDdb1 Promotes Muscle Atrophy through Encoding a Novel Protein CircDdb1-867aa. Adv Sci (Weinh). 2024;11:e2406986.

**2. Supplementary Figures and Legends**

**
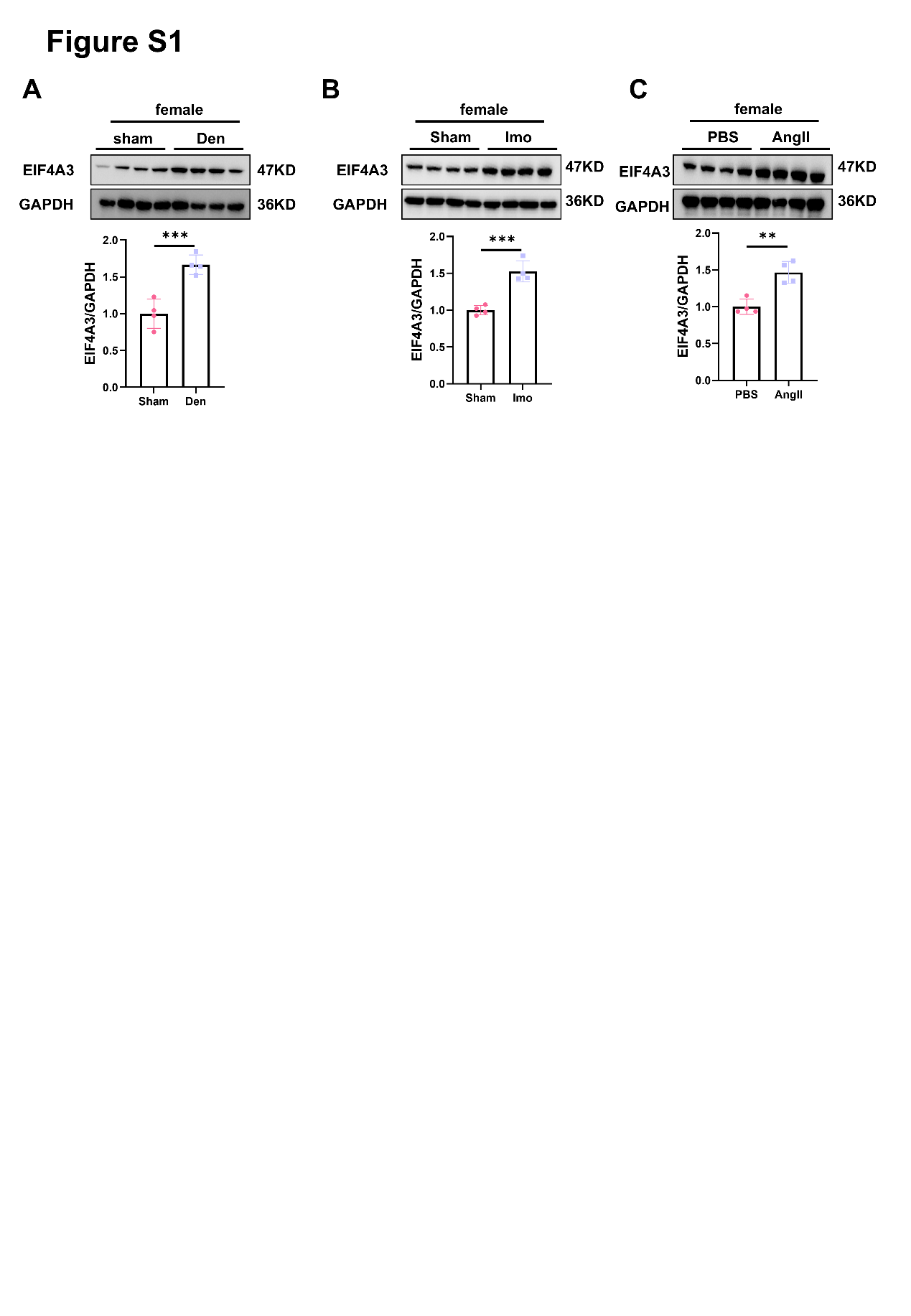
**

**Figure S1 Expression of EIF4A3 in atrophic muscle in female mice.**

Western blot analysis of EIF4A3 expression levels in gastrocnemius muscle tissues of denervation (Den)- (A), immobilization (Imo)- (B), and Angiotensin II (Ang II)- (C) induced muscle atrophy models in female mice (n=4 per group). The comparison between two groups was performed using Student’s t-test. The statistical results were represented by Mean±SD. **p < 0.01, ***p < 0.001.


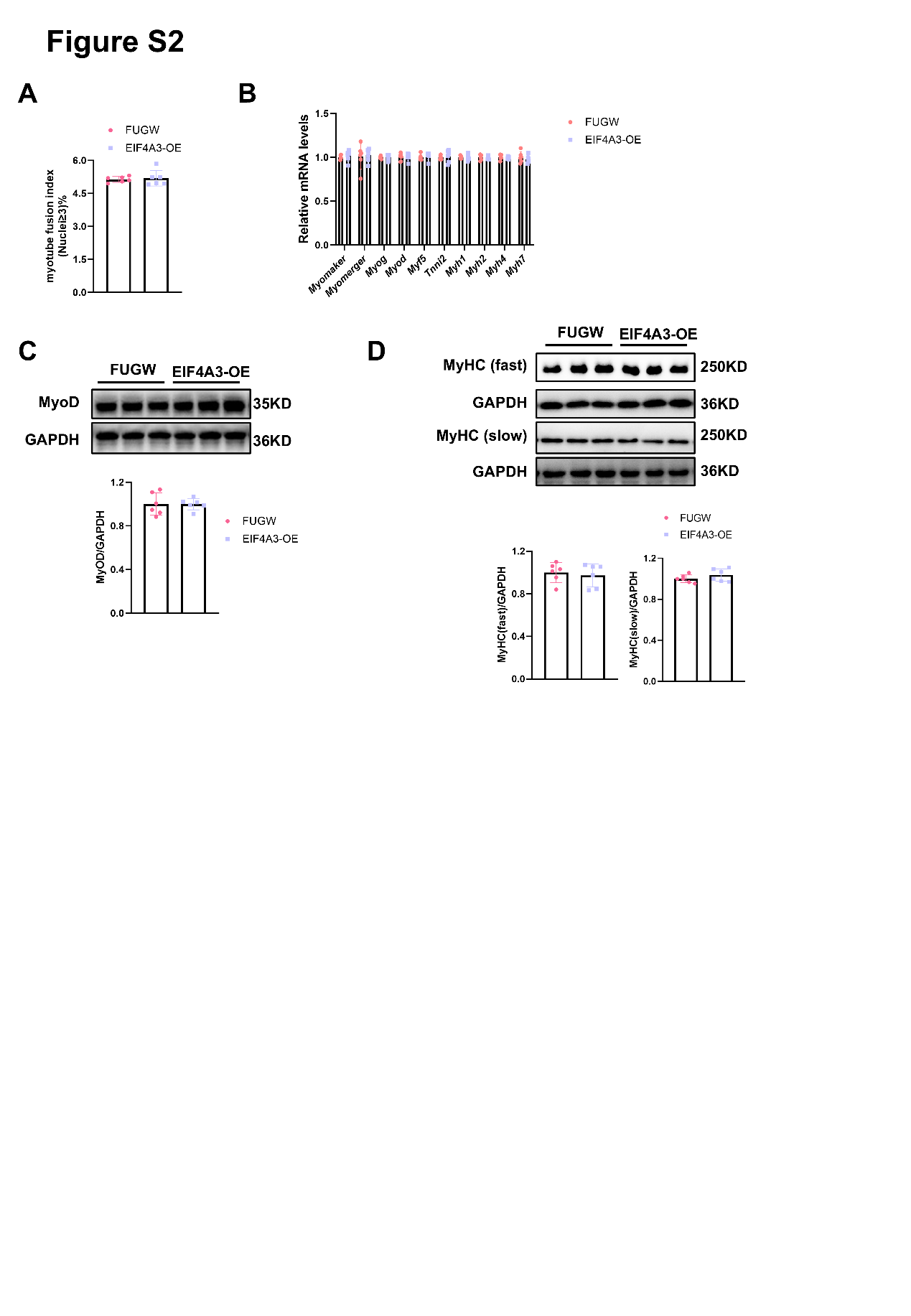
**Figure S2 EIF4A3 overexpression doesn’t affect the myogenic differentiation and fast- and slow-type isoforms in mice myotube cells.**

(A) The myotube fusion index of C2C12 myotubes transfected with EIF4A3-OE for 2 days (n = 4 per group). (B) Analysis of myogenic differentiation-associated genes and fast- and slow-type isoform-associated genes in C2C12 myotubes transfected with EIF4A3-OE and FUGW lentivirus were evaluated by RT-qPCR (n=6). (C and D) Western blot analysis of MyoD, fast or slow myosin heavy chain (MyHC) protein expression levels in C2C12 myotubes transfected with EIF4A3-OE and control lentivirus (n=6). The comparison between two groups was performed using Student’s t-test. The statistical results were represented by Mean±SD.


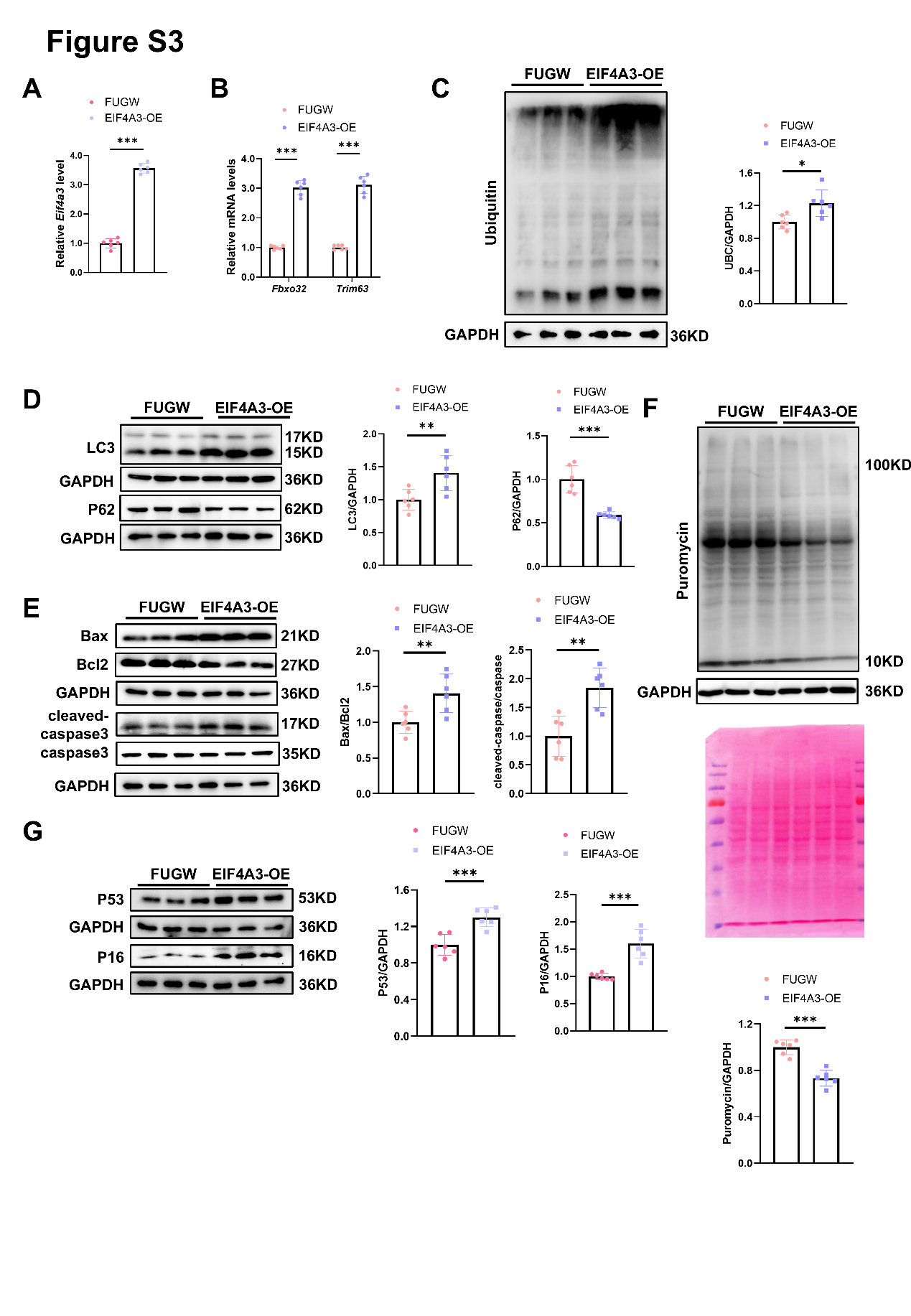
**Figure S3 EIF4A3 promotes muscle atrophy and muscle aging *in vitro*.**

(A) Expression levels of ***Eif4a3*** in C2C12 myotubes transfected with EIF4A3 overexpression (EIF4A3-OE) and controls (FUGW) lentivirus were evaluated by RT-qPCR (n=6). **(B)** **Expression levels of** ***Fbxo32* and *Trim63* genes in C2C12 myotube transfected with EIF4A3-OE and controls lentivirus were evaluated by RT-qPCR (n=6).** (C) Western blot analysis of ubiquitin-protein expression in C2C12 myotubes transfected with EIF4A3-OE and control lentivirus (n=6). (D) Western blot analysis of P62 and LC3 protein expression levels in C2C12 myotubes transfected with EIF4A3-OE and control lentivirus (n=6). (E) Western blot analysis of caspase 3 and Bax/Bcl_2_ protein expression levels in C2C12 myotubes transfected with EIF4A3-OE and control lentivirus (n=6). (F) Western blot analysis of protein synthesis by anti-Puromycin in C2C12 myotubes transfected with EIF4A3-OE and control lentivirus (n=6). (G) Western blot analysis of expression levels of aging marker proteins P53 and P16 in C2C12 myotubes transfected with EIF4A3-OE and control lentivirus (n=6). The comparison between two groups was performed using Student’s t-test. The statistical results were represented by Mean±SD. *p < 0.05, **p < 0.01, ***p < 0.001.

**
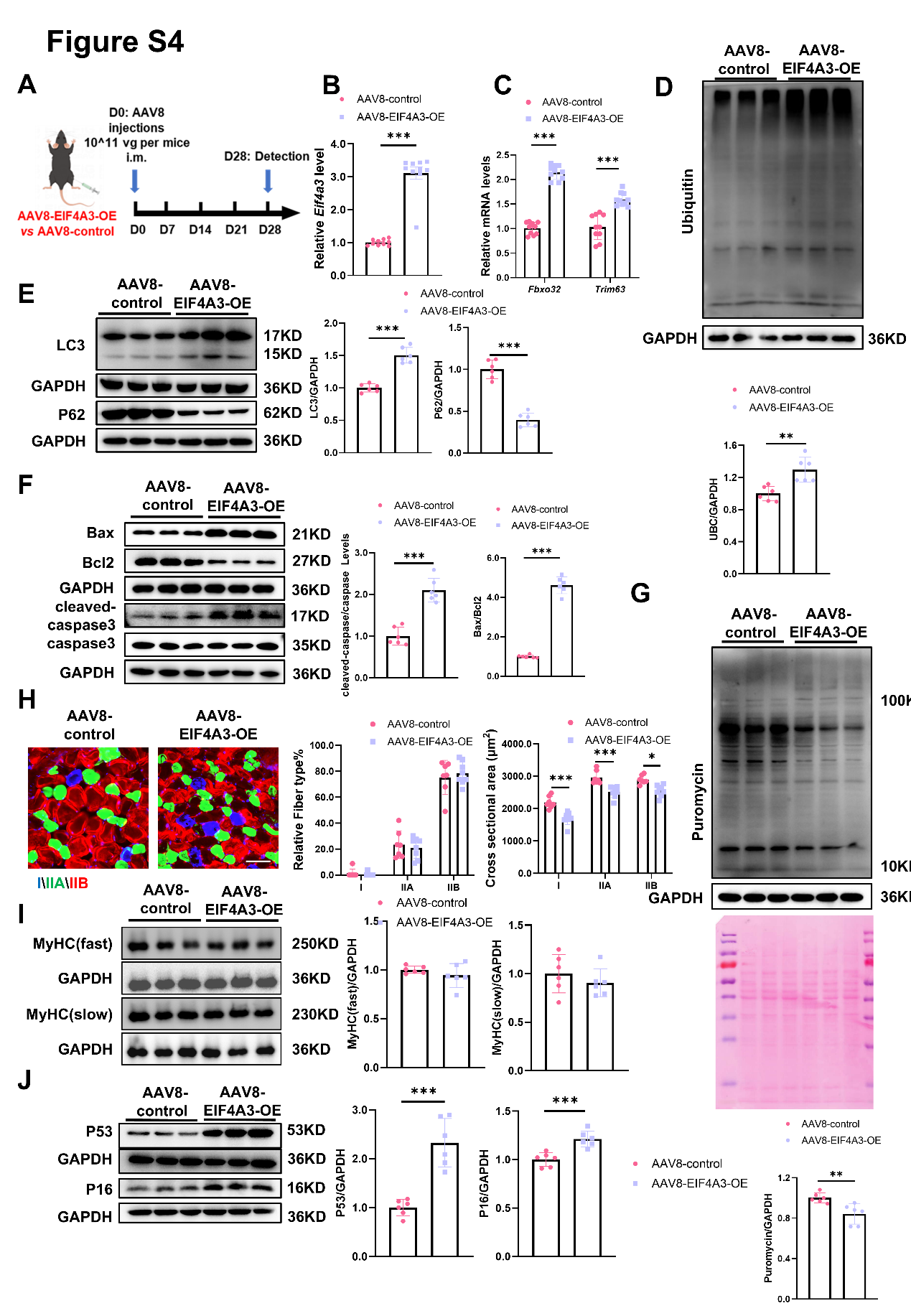
Figure S4 EIF4A3 induces muscle atrophy *in vivo*.**

(A) Schematic design of the experiment including the viral injection dose and the experimental time point. (B) Expression level of ***Eif4a3*** in gastrocnemius of mice injected with AAV8-EIF4A3-OE and AAV8-control evaluated by RT-qPCR (n=10). **(C) Analysis of** ***Fbxo32* and *Trim63* mRNA levels in gastrocnemius of mice injected with AAV8-EIF4A3-OE and AAV8-control evaluated by RT-qPCR (n=10).** (D) Western blot analysis of ubiquitin-protein expression in gastrocnemius of mice injected with AAV8-EIF4A3-OE and AAV8- controls (n=6). (E) Western blot analysis of P62 and LC3 protein expression levels in gastrocnemius of mice injected with AAV8-EIF4A3-OE and AAV8- controls (n=6). (F) Western blot analysis of caspase3 and Bax/Bcl_2_ protein expression levels in gastrocnemius of mice injected with AAV8-EIF4A3-OE and AAV8-control (n=6). (G) Western blot analysis of protein synthesis by anti- Puromycin in muscle of mice injection with AAV8-EIF4A3-OE and AAV8- control (n=6). (H) The types of gastrocnemius fibers in mice injected with AAV8-EIF4A3-OE and AAV8-control were detected by immunofluorescence staining (n=7 in AAV8-control group, n=8 in AAV8-EIF4A3-OE group). (I) Western blot analysis of fast or slow myosin heavy chain (MyHC) protein expression levels in muscle of mice injection with AAV8-EIF4A3-OE and AAV8- control (n=6). (J) Western blot analysis of expression levels of aging marker proteins P53 and P16 in gastrocnemius of mice injected with AAV8-EIF4A3-OE and AAV8-control (n=6). The comparison between two groups was performed using Student’s t-test. The statistical results were represented by Mean±SD. *p < 0.05, **p < 0.01, ***p < 0.001.


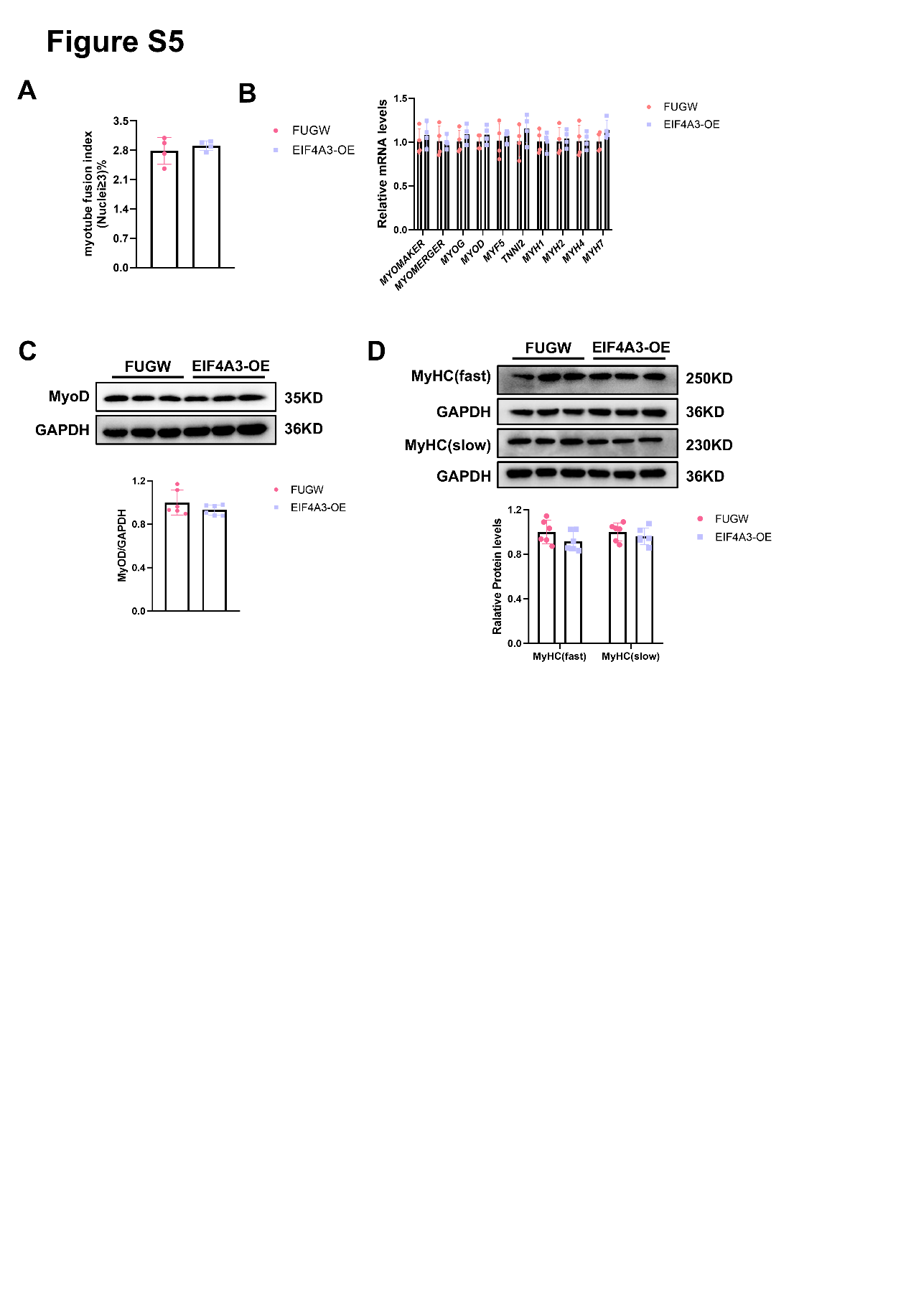
**Figure S5 EIF4A3 overexpression doesn’t affect the myogenic differentiation and fast- and slow-type isoforms in human myotube cells.**

(A) The myotube fusion index of human myotubes transfected with EIF4A3-OE for 2 days (n = 4 per group). (B) Analysis of myogenic differentiation-associated genes and fast- and slow-type isoform-associated genes in human myotubes transfected with EIF4A3-OE and FUGW lentivirus were evaluated by RT-qPCR (n=4). (C and D) Western blot analysis of MyoD, fast or slow myosin heavy chain (MyHC) protein expression levels in human myotubes transfected with EIF4A3-OE and control lentivirus (n=6). The comparison between two groups was performed using Student’s t-test. The statistical results were represented by Mean±SD.

**
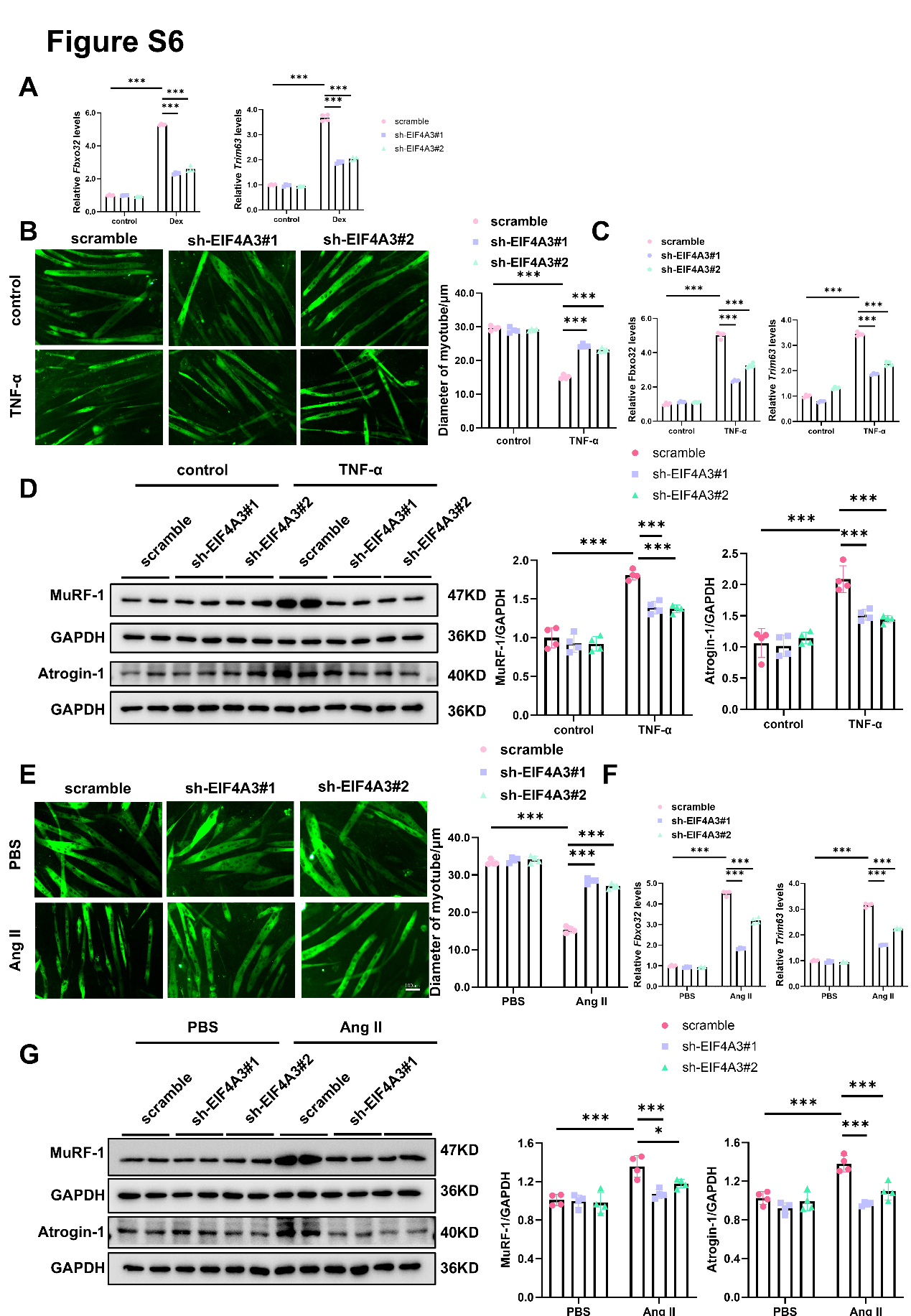
**

**Figure S6 Inhibition of EIF4A3 expression prevents muscle atrophy *in vitro*.**

**(A)Expression of** ***Fbxo32* and *Trim63* in C2C12 myotubes transfected with sh-EIF4A3#1 and sh-EIF4A3#2 lentivirus in Dex-induced myotube atrophy model was analyzed by RT-qPCR (n=4).** (B) Immunofluorescence staining and quantification of C2C12 myotubes diameter transfected with sh-EIF4A3#1 and sh-EIF4A3#2 lentivirus in a TNF-α-induced muscular atrophy model. (n=4; Scale :100μm). **(C) *Fbxo32* and *Trim63* expression in C2C12 myotubes transfected with sh-EIF4A3#1 and sh-EIF4A3#2 lentivirus in a TNF-α-induced muscle atrophy model was analyzed by RT-qPCR (n=4).** (D) **MuRF-1 and** **Atrogin1** expression in C2C12 myotubes transfected with sh-EIF4A3#1 and sh-EIF4A3#2 lentivirus in a TNF-α-induced muscle atrophy model was analyzed by western blot (n=4). (E) Immunofluorescence staining and quantification of C2C12 myotubes diameter transfected with sh-EIF4A3#1 and sh-EIF4A3#2 lentivirus in Ang II-induced muscle atrophy models. (n=4; Scale :100 μm).**(F)** ***Fbxo32* and *Trim63* expression in C2C12 myotubes transfected with sh-EIF4A3#1 and sh-EIF4A3#2 lentivirus in a Ang II-induced muscle atrophy model was analyzed by RT-qPCR (n=4).** (G) **MuRF-1 and** **Atrogin1** expression in C2C12 myotube transfected with sh-EIF4A3#1 and sh-EIF4A3#2 lentivirus in Ang II-induced muscle atrophy models was analyzed by western blots (n=4). Multiple group comparisons were conducted using one-way or two-way ANOVA, followed by Dunnett’s T3 or Bonferroni post-hoc tests, depending on the homogeneity of variance assessed. The statistical results were represented by Mean±SD. ***p < 0.001.

**
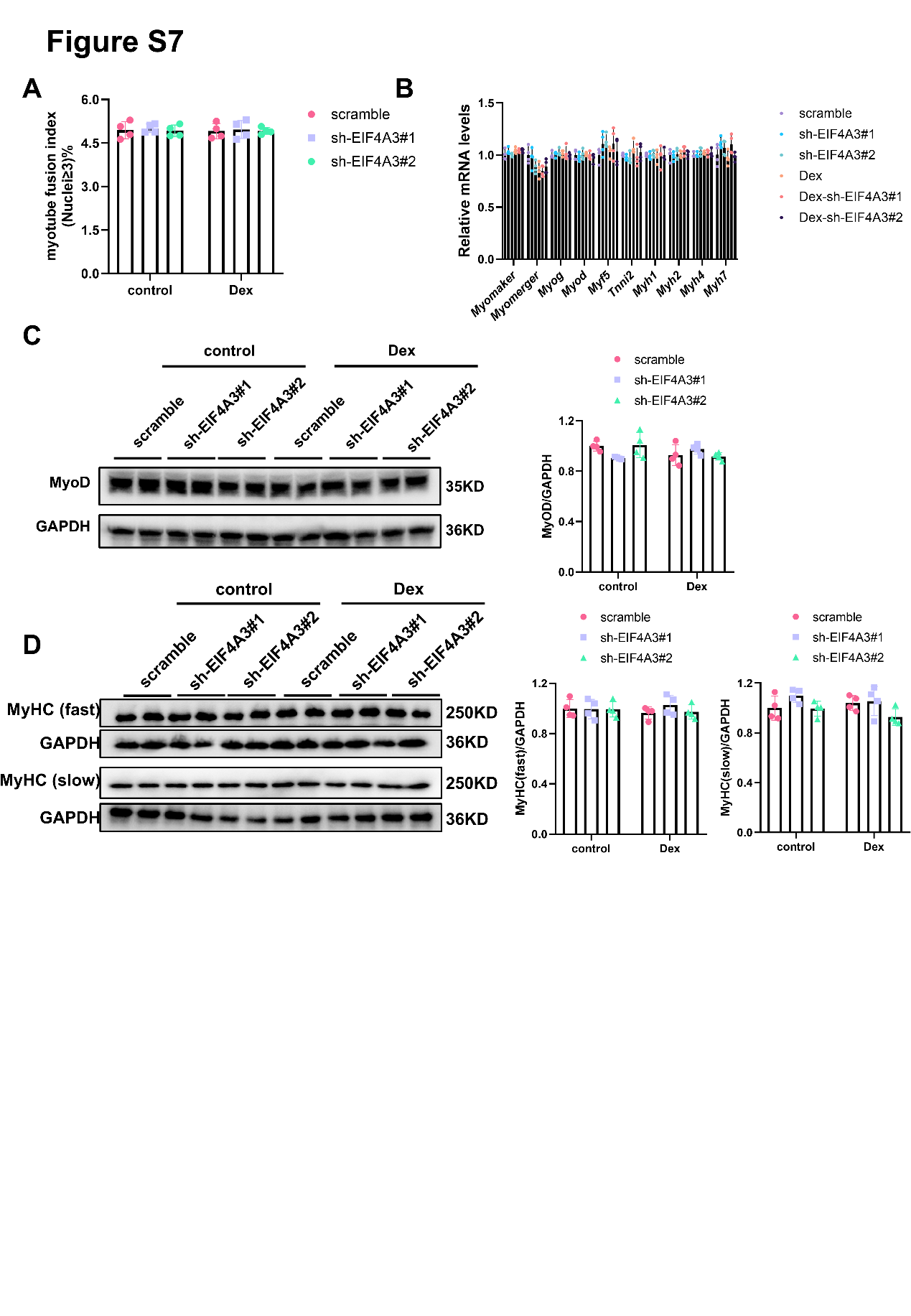
**

**Figure S7** **Inhibition of EIF4A3 expression doesn’t affect the myogenic differentiation and fast- and slow-type isoforms during dexamethasone treatment.**

(A) The myotube fusion index of C2C12 myotubes transfected with sh-EIF4A3#1 and sh-EIF4A3#2 lentivirus in a dexamethasone (Dex) -induced myotube (n = 4 per group). (B) Analysis of myogenic differentiation-associated genes and fast- and slow-type isoform-associated genes in C2C12 myotubes transfected with sh-EIF4A3#1 and sh-EIF4A3#2 lentivirus in a dexamethasone (Dex) -induced myotube (n=4). (C and D) Western blot analysis of MyoD, fast or slow myosin heavy chain (MyHC) protein expression levels in C2C12 myotubes transfected with sh-EIF4A3#1 and sh-EIF4A3#2 lentivirus in a dexamethasone (Dex) -induced myotube (n=4). followed by Dunnett’s T3 or Bonferroni post-hoc tests, depending on the homogeneity of variance assessed. The statistical results were represented by Mean±SD.

**
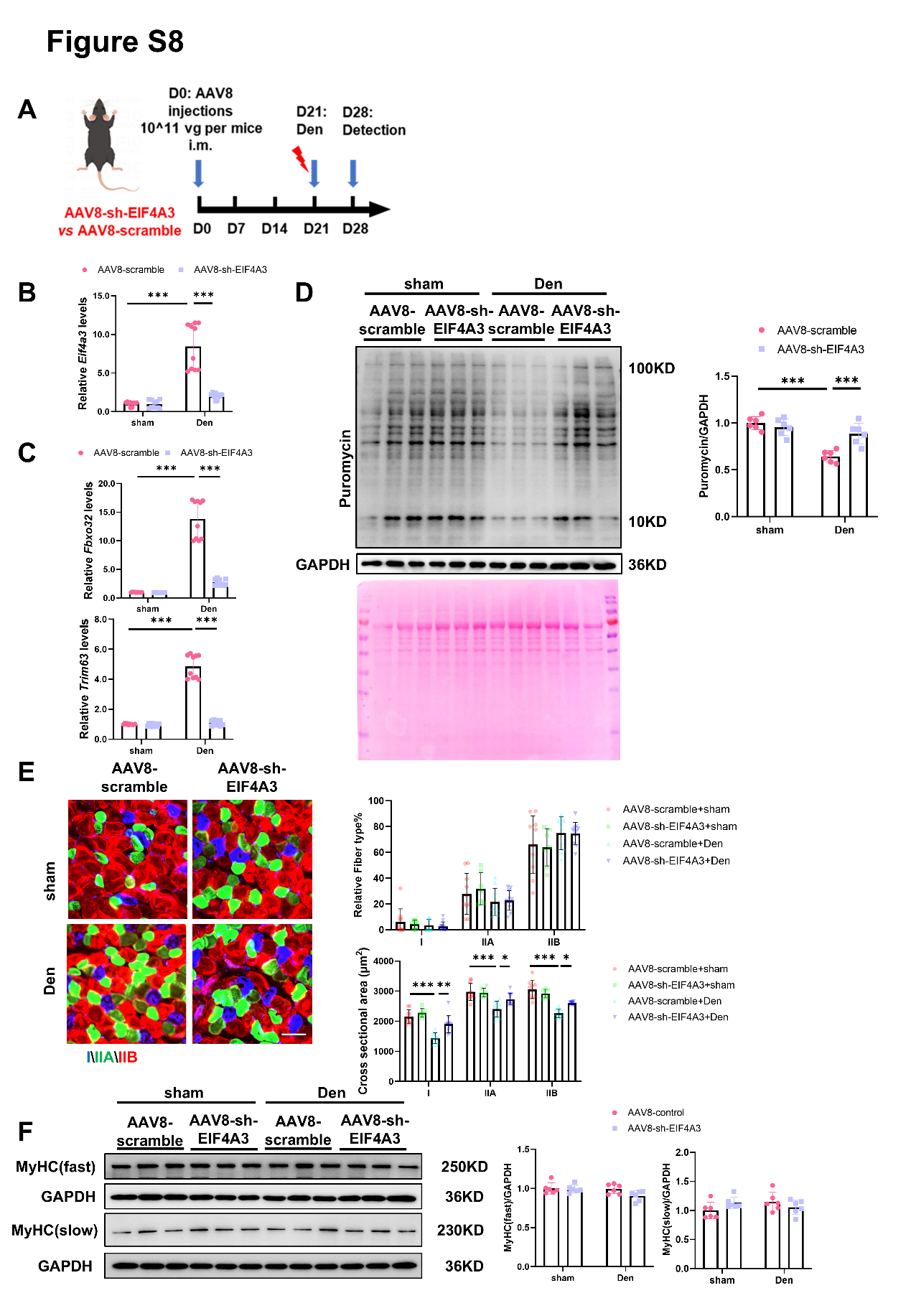
**

**Figure S8 Inhibition of EIF4A3 expression prevents muscle atrophy caused by denervation *in vivo*.**

(A) Experimental design process and schematic diagram of virus injection dose.

(B)The expression level of ***Eif4a3*** in gastrocnemius of AAV8-sh-EIF4A3 and AAV8-scramble mice was detected by RT-qPCR (n=10). **(C) The expression level of** ***Fbxo32* and *Trim63* in gastrocnemius of AAV8-sh-EIF4A3 and AAV8-scramble mice was detected by RT-qPCR (n=10).** (D) Western blot analysis of protein synthesis by anti-Puromycin in muscle of mice injected with AAV8-sh-EIF4A3 and AAV8-scramble (n=6). (E) The types of gastrocnemius fibers in muscle of mice injected with AAV8-sh-EIF4A3 and AAV8-scramble were detected by immunofluorescence staining (n=7-11). (F) Western blot analysis of fast or slow myosin heavy chain (MyHC) protein expression levels in muscle of mice injection with AAV8-sh-EIF4A3 and AAV8-scramble during denervation (n=6). Multiple group comparisons were conducted using one-way or two-way ANOVA, followed by Dunnett’s T3 or Bonferroni post-hoc tests, depending on the homogeneity of variance assessed. The statistical results were represented by Mean±SD. ***p < 0.001.

**
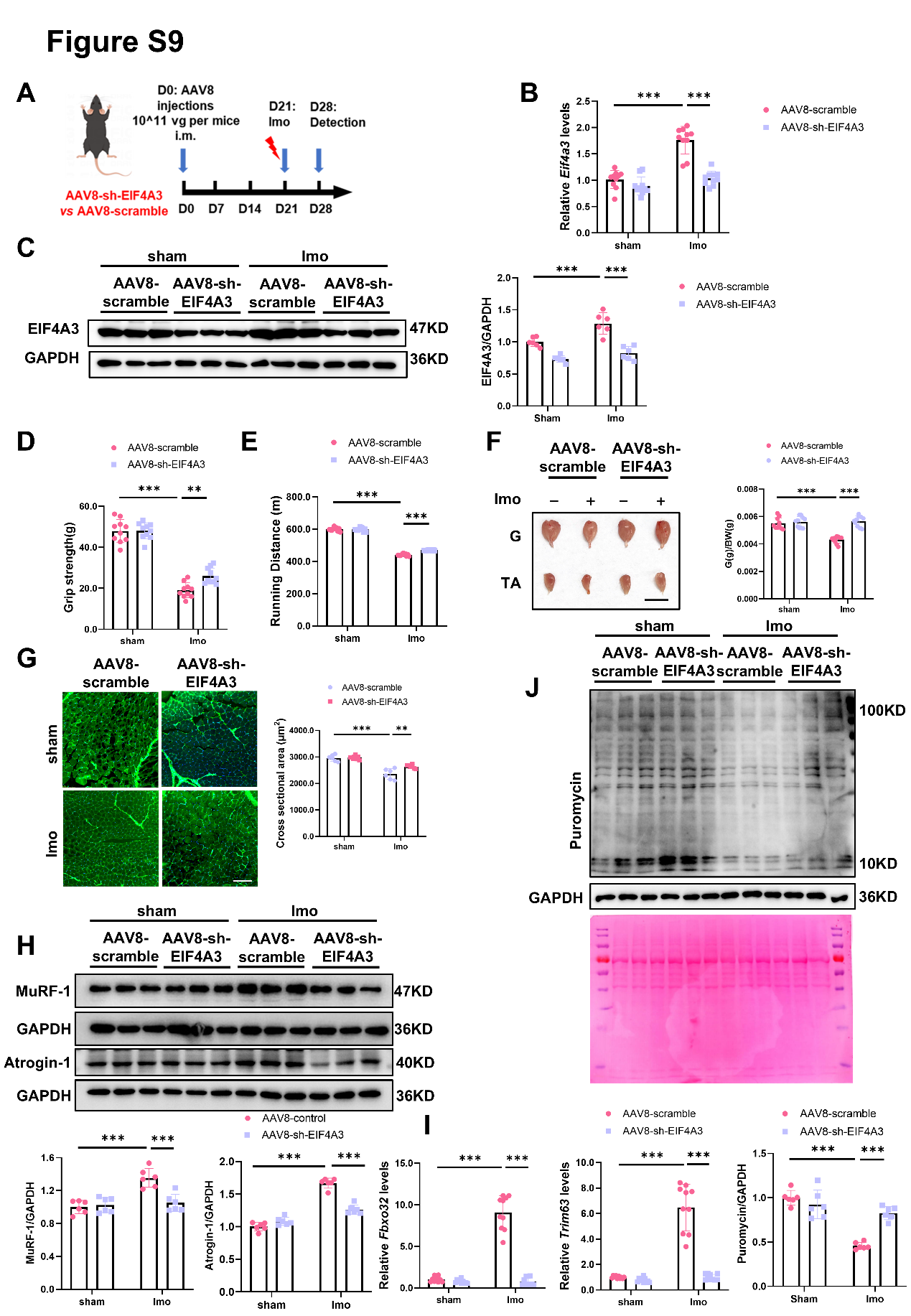
**

**Figure S9 Inhibition of EIF4A3 expression prevents muscle atrophy caused by** **immobilization *in vivo*.**

(A) Experimental design process and schematic diagram of virus injection dose. (B) The expression level of ***Eif4a3*** in the gastrocnemius of AAV8-sh-EIF4A3 and AAV8-scramble mice was detected by RT-qPCR (n=10). (C) Western blot analysis of EIF4A3 protein expression in gastrocnemius of mice injected with AAV8-sh-EIF4A3 and AAV8-scramble (n=6). (D) Statistical analysis of grip strength of right hind limb muscles in mice injected with AAV8-sh-EIF4A3 and AAV8-scramble (n=10). (E) Running distance of mice injected with AAV8-sh-EIF4A3 and AAV8-scramble (n=10). (F) Representative images of morphology and body weight (G/BW) of gastrocnemius in mice injected with AAV8-sh-EIF4A3 and AAV8-scramble (n=10, scale: 1 cm). (G) Representative images and statistical analysis of muscle fiber cross-sectional area in mice injected with AAV8-sh-EIF4A3 and AAV8-scramble (n =6,8,6,6; Bar: 50μm). (H) Expression levels of **MuRF-1 and Atrogin-1** in gastrocnemius of mice injected with AAV8-sh-EIF4A3 and AAV8-scramble were detected by western blot (n=6). **(I) Expression levels of** ***Fbxo32* and *Trim63* genes in gastrocnemius of mice injected with AAV8-sh-EIF4A3 and AAV8-scramble were detected by RT-qPCR (n=10).** (J)Western blot analysis of protein synthesis by anti-Puromycin in gastrocnemius of mice injected with AAV8-sh-EIF4A3 and AAV8-scramble (n=6). Multiple group comparisons were conducted using one-way or two-way ANOVA, followed by Dunnett’s T3 or Bonferroni post-hoc tests, depending on the homogeneity of variance assessed. The statistical results were represented by Mean±SD. **p < 0.01, ***p < 0.001.

**
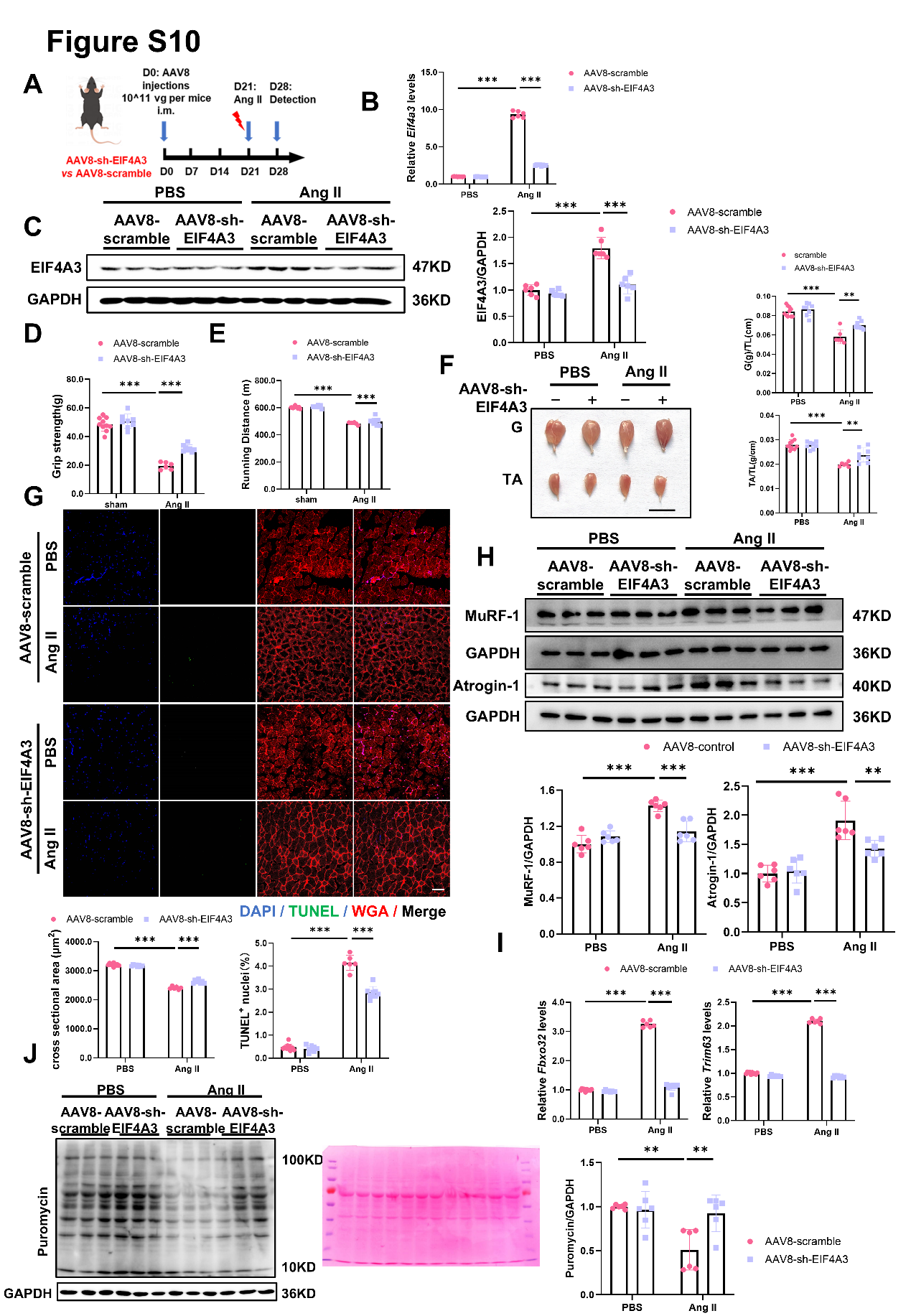
**

**Figure S10 Inhibition of EIF4A3 expression prevents angiotensin II-induced muscle atrophy *in vivo*.**

(A) Experimental design process and schematic diagram of virus injection dose. (B) The expression level of ***Eif4a3*** gene in the gastrocnemius of AAV8-sh-EIF4A3 and AAV8-scramble mice was detected by RT-qPCR (n=10,8,6,8). (C) Western blot analysis of the expression of EIF4A3 protein in gastritis muscle of mice injected with AAV8-sh-EIF4A3 and AAV8-scramble (n=6). (D) Statistical analysis of grip strength in right hind limb muscles of mice injected with AAV8-sh-EIF4A3 and AAV8-scramble (n=10,8,6,8). (E) Running distance of mice injected with AAV8-sh-EIF4A3 and AAV8-scramble (n=10,8,6,8). (F) Representative images of morphology and the ratio of skeletal muscle (Gastrocnemius muscle, G; Tibialis anterior muscle, TA) weight and tibial length (TL) of gastrocnemius muscle in mice injected with AAV8-sh-EIF4A3 and AAV8-scramble (n=10, 8, 6, 8, scale: 1 cm). (G) Representative images and statistical analysis of muscle fiber cross-sectional area, and TUNEL staining in mice injected with AAV8-sh-EIF4A3 and AAV8-scramble (n=10,8,6,8; Bar: 50μm). (H) Expression levels of **MuRF-1 and Atrogin-1** protein in gastrocnemius of mice injected with AAV8-sh-EIF4A3 and AAV8-scramble were detected by western blot (n=10,8,6,8). **(I) The expression level of** ***Fbxo32* and *Trim63* gene in the gastrocnemius of AAV8-sh-EIF4A3 and AAV8-scramble mice was detected by RT-qPCR (n=10,8,6,8).** (J) Western blot analysis of protein synthesis by anti-Puromycin in gastrocnemius of mice injected with AAV8-sh-EIF4A3 and AAV8-scramble (n=6). Multiple group comparisons were conducted using one-way or two-way ANOVA, followed by Dunnett's T3 or Bonferroni post-hoc tests, depending on the homogeneity of variance assessed. The statistical results were represented by Mean±SD. **p < 0.01, ***p < 0.001.

**
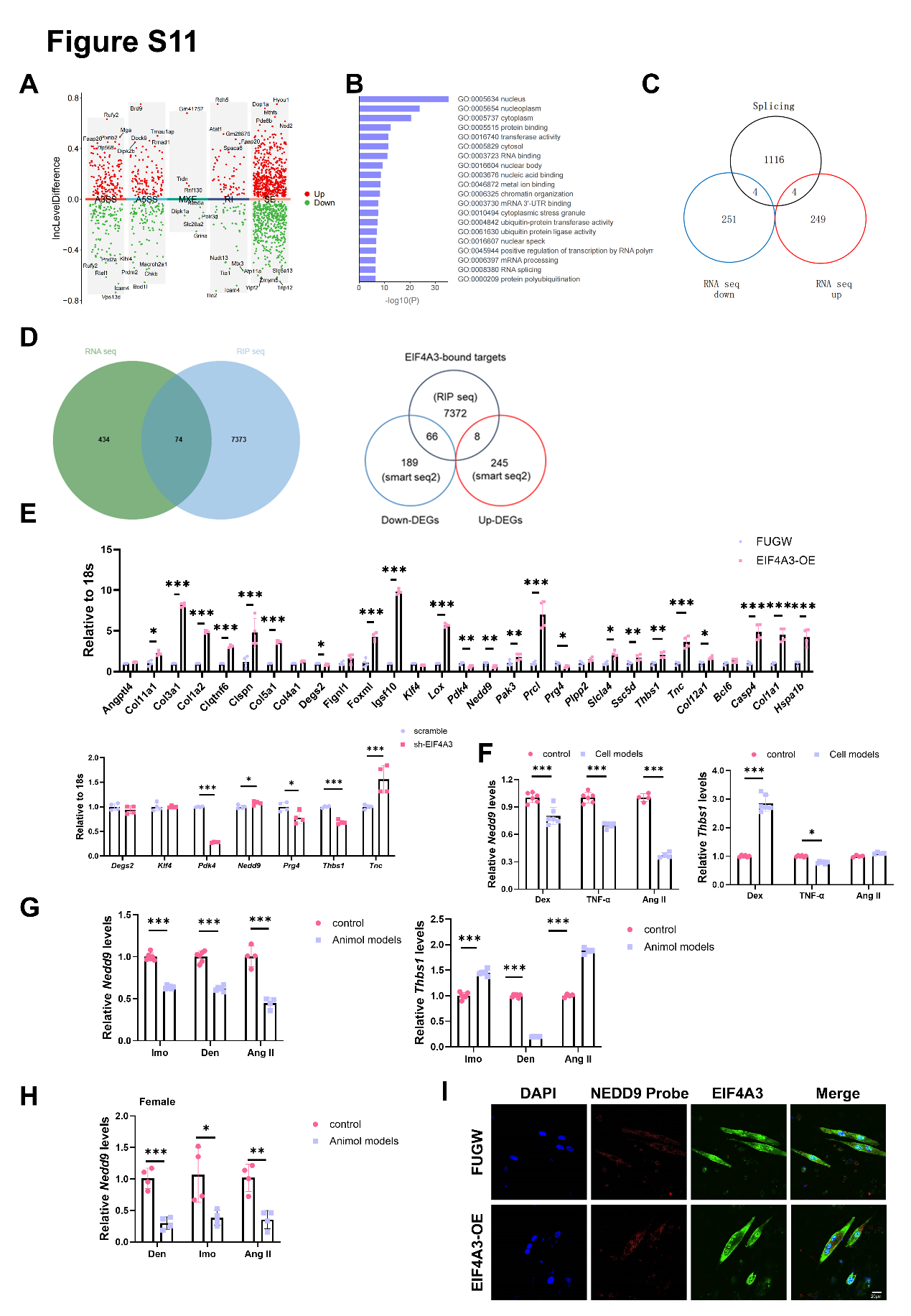
**

**Figure S11 Filter out NEDD9 mRNA to be the downstream of EIF4A3.**

(A) Volcanoplots showing inclusion level differences in various ASEs between EIF4A3 overexpression and controls. (B) Bar graphs showing the enriched terms across splicing-altered genes. (C) Venn diagrams showing the overlap between dysregulated genes and genes with altered splicing in EIF4A3 overexpression. (D) Venn diagrams diagram for the overlap between dysregulated genes and RNAs binding with EIF4A3. And Venn diagrams showing the overlap between dysregulated genes in EIF4A3 overexpression and EIF4A3 binding mRNA. (E) Differential genes were screened by C2C12 myotubes treated with EIF4A3-OE and sh-EIF4A3 lentivirus (n=4). Further screening was performed by (F) cell muscle atrophy models (n=4) and (G) mice muscle atrophy models (n=4). (H) RT-qPCR analysis of *Nedd9* expression levels in gastrocnemius muscle tissues of mice muscle atrophy models in female mice (n=4 per group). (I) Subcellular distribution of *Nedd9* mRNA and EIF4A3 in baseline and EIF4A3 OE treated myotube. The comparison between two groups was performed using Student’s t-test. *p < 0.05, **p < 0.01, ***p < 0.001.

**
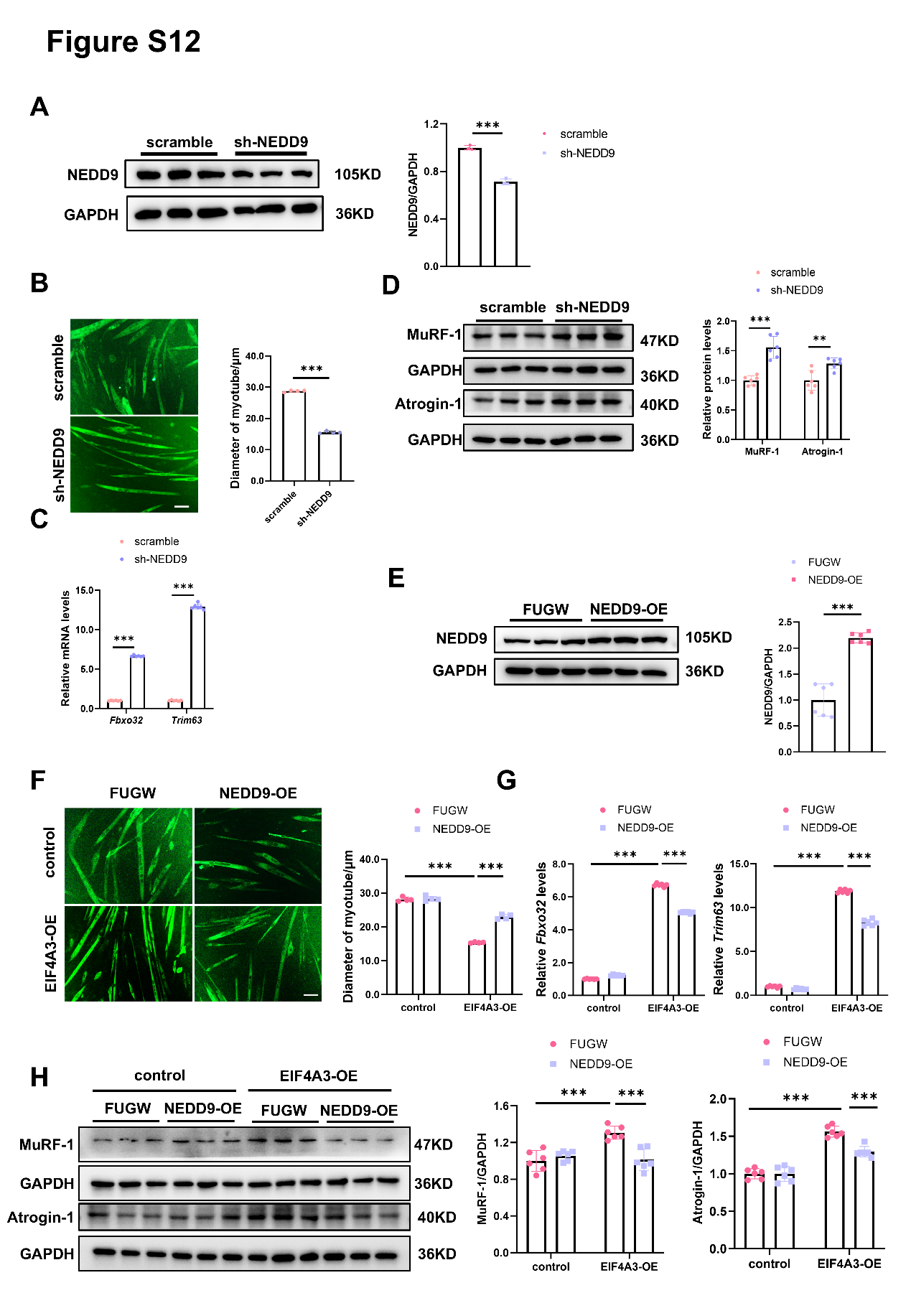
**

**Figure S12 EIF4A3 facilitates muscle atrophy via binding the NEDD9 mRNA and promoting RNA degradation.**

(A)Western Blot was used to detect the expression level of NEDD9 in C2C12 myotubes transfected with sh-NEDD9 lentivirus (n=6). (B) Representative images and statistical analysis of C2C12 myotubes transfected with sh-NEDD9 and control lentivirus (n=4), scale: 100μm. **(C) Expression levels of** ***Fbxo32* and *Trim63* genes in C2C12 myotube transfected with sh-NEDD9 and controls lentivirus were evaluated by RT-qPCR (n=6).** (D)Expression levels of **MuRF-1 and Atrogin-1** proteins in C2C12 myotube transfected with sh-NEDD9 and controls lentivirus were evaluated by western blot (n=6). (E)Western Blot was used to detect the expression level of NEDD9 in C2C12 myotubes transfected with NEDD9-OE lentivirus (n=6). (F) The changes of myotubes’ diameter in C2C12 myotubes treated with EIF4A3-OE and NEDD9-OE lentivirus were detected by immunofluorescence (n=4). **(G) The expression levels of *Fbxo32* and *Trim63* genes in C2C12 myotubes treated with EIF4A3-OE and NEDD9-OE lentivirus were detected by RT-qPCR (n=6).** (H) The expression levels of **MuRF-1 and Atrogin-1** proteins in C2C12 myotubes treated with EIF4A3-OE and NEDD9-OE lentivirus were detected by western blot (n=6). The comparison between two groups was performed using Student’s t-test (A-E). Multiple group comparisons were conducted using one-way or two-way ANOVA, followed by Dunnett’s T3 or Bonferroni post-hoc tests, depending on the homogeneity of variance assessed (F-H). The statistical results were represented by Mean±SD. ***p < 0.001.

**
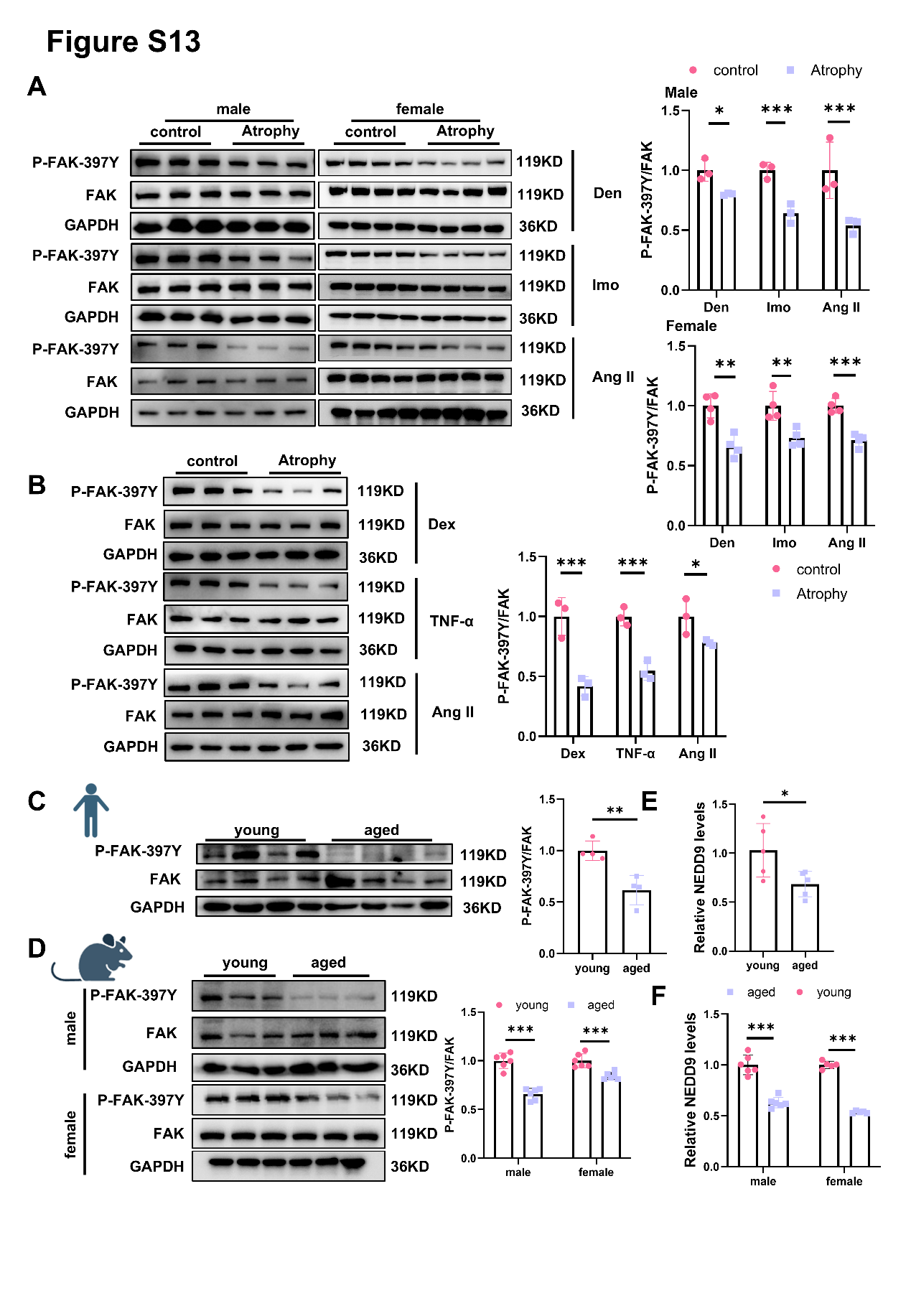
**

**Figure S13 Inactivation of NEDD9-FAK pathway in muscle atrophy model and muscle aging.**

Western blot analysis of FAK pathway expression in (A) muscle atrophy model (n=3 in male, n=4 in famale) and C2C12 cell atrophy model (B) (n=3). Western blot analysis of FAK pathway protein expression in (C) aged human muscle (n=4) and (D) aged mouse gastrocnemius tissue (n=6 in male, n=6 in female). RT-qPCR analysis of NEDD9 mRNA expression in (E) aged human muscle (n=5) and (F) aged mouse gastrocnemius tissue (n=6 in male, n=6 in female). The comparison between two groups was performed using Student’s t-test. The statistical results were represented by Mean±SD. *p < 0.05, **p < 0.01, ***p < 0.001.

**
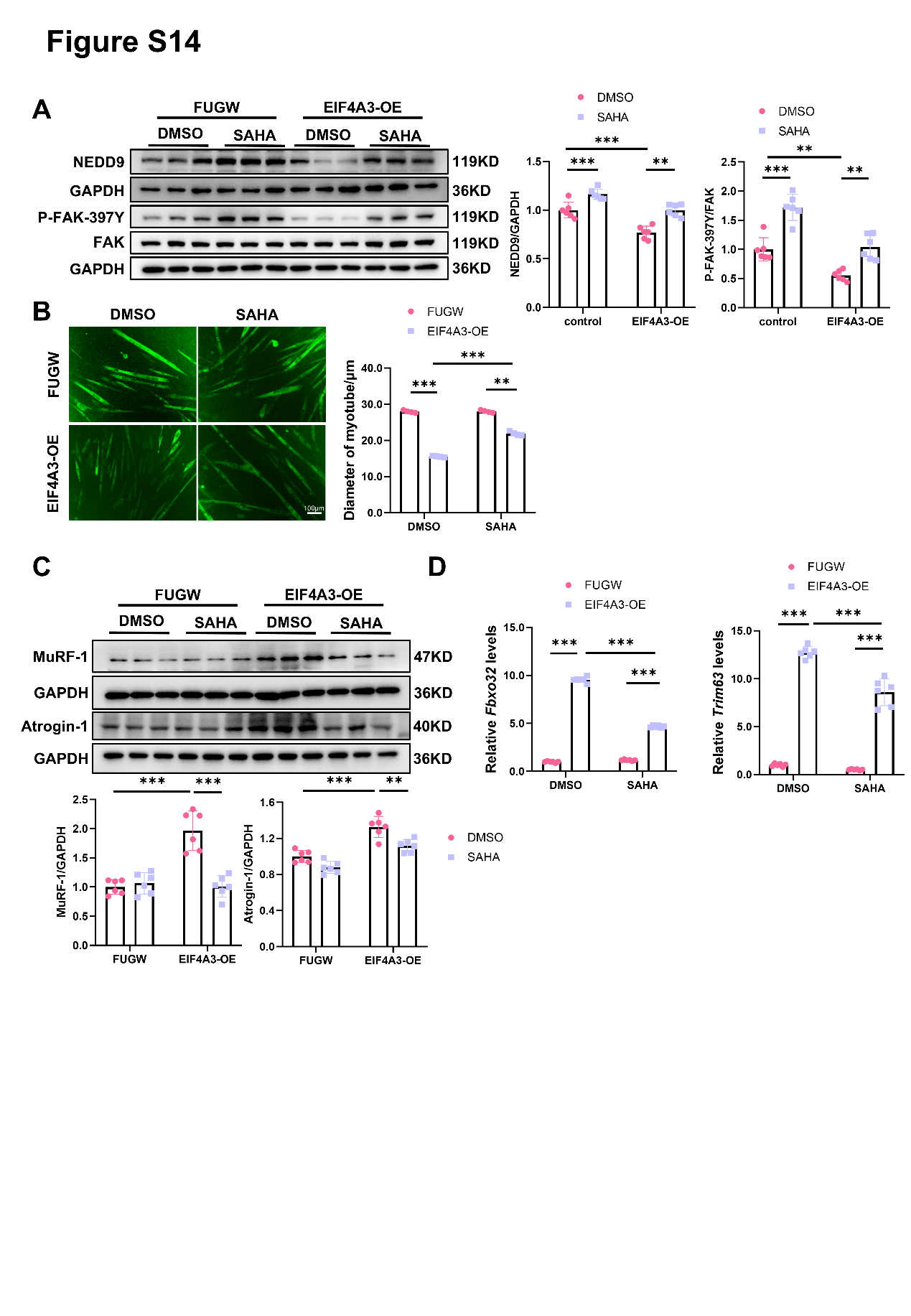
**

**Figure S14 SAHA stimulation of NEDD9-FAK signaling pathway can alleviate muscle atrophy induced by EIF4A3 overexpression *in vitro*.**

(A) Western Blot analysis of the expression levels of NEDD9 and FAK pathway proteins in C2C12 myotubes treated with EIF4A3-OE lentivirus and SAHA (n=6). (B) The change of myotubes’ diameter of C2C12 myotube after treatment of EIF4A3-OE lentivirus and SAHA was detected by immunofluorescence (n=4). (C) The expression levels of **MuRF-1 and Atrogin-1** proteins in C2C12 myotubes after transfection with EIF4A3-OE and SAHA were detected by western blot (n=6). **(D) The expression levels of** ***Fbxo32* and *Trim63* genes in C2C12 myotubes after transfection with EIF4A3-OE and SAHA were detected by RT-qPCR (n=6).** Multiple group comparisons were conducted using one-way or two-way ANOVA, followed by Dunnett’s T3 or Bonferroni post-hoc tests, depending on the homogeneity of variance assessed. The statistical results were represented by Mean±SD. **p < 0.01, ***p < 0.001.

**
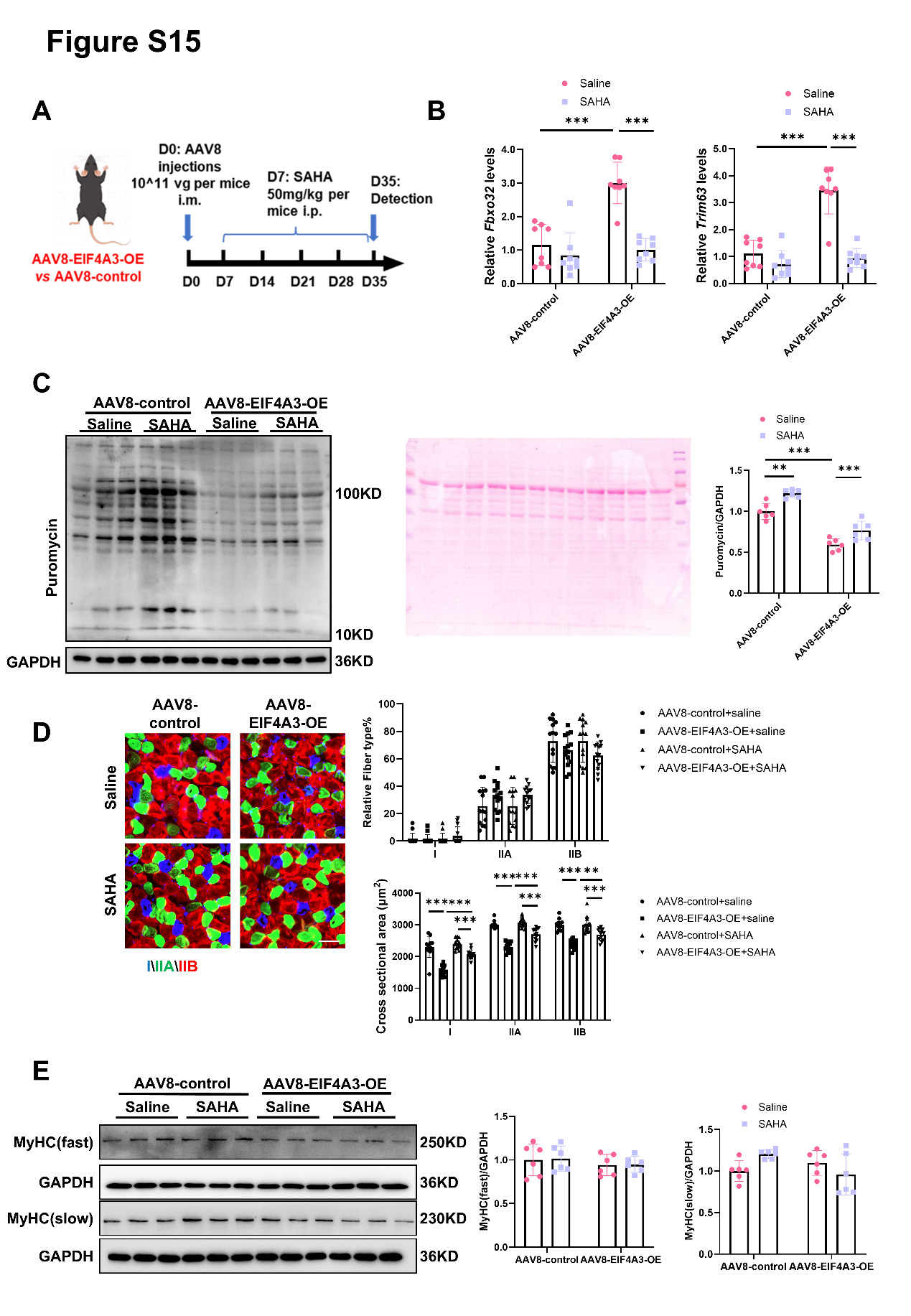
**

**Figure S15 SAHA stimulation of NEDD9-FAK signaling pathway can alleviate muscle atrophy induced by EIF4A3 overexpression *in vivo*.**

(A) Experimental design process and schematic diagram of virus injection dose. **(B) Expression levels of *Fbxo32* and *Trim63* genes in gastrocnemius of mice injected with AAV8-EIF4A3-OE and SAHA were detected by RT-qPCR (n=8).** (C)Western blot analysis of Puromycin protein expression in gastrocnemius of mice injected with AAV8-EIF4A3-OE and SAHA (n=6). (D) The types of gastrocnemius fibers in muscle of mice injected with AAV8-EIF4A3-OE and SAHA were detected by immunofluorescence staining (n=12-14). (E) Western blot analysis of fast or slow myosin heavy chain (MyHC) protein expression levels in muscle of mice mice injected with AAV8-EIF4A3-OE and SAHA (n=6). Multiple group comparisons were conducted using one-way or two-way ANOVA, followed by Dunnett’s T3 or Bonferroni post-hoc tests, depending on the homogeneity of variance assessed. The statistical results were represented by Mean±SD. **p < 0.01, ***p < 0.001.

**Table S1 primers used in this study**

| Gene Name | forward primer (5’-3’) | reverse primer(5’-3’) |
| --- | --- | --- |
| *mmu-18s* | TCAAGAACGAAAGTCGGAGG | GGACATCTAAGGGCATCAC |
| *mmu-Eif4a3* | GCTGGACTACGGACAGCAC | CTTCCGCTTGGTGTTGCAGA |
| *mmu-Nedd9* | TCAAGTGCCAAATTCCCAGG | GTGCCGCCAATGTTCCTCT |
| *hsa-NEDD9* | ATGGCAAGGGCCTTATATGACA | TTCTGCTCTATGACGGTCAGG |
| *mmu-Fbxo32* | CAGCTTCGTGAGCGACCTC | GGCAGTCGAGAAGTCCAGTC |
| *mmu-Trim63* | GTGTGAGGTGCCTACTTGCTC | GCTCAGTCTTCTGTCCTTGGA |
| *mmu-Myomerger* | CCAGAAGAAAGCTGCACTGTAAA | GAGCCTCTCTCATGTCTTGGG |
| *mmu-Myomaker* | GAGCATCGCTACCAAGAGGC | GAATGTCACGGCGCATGAAG |
| *mmu-Tnni2* | AAAGCCGCCGAGAATCTGAG | TCGCATGCAGTTGTTTGCAG |
| *mmu-Myod* | TGAATGAGGCCTTCGAGACG | GCCTGCAGACCTTCGATGTA |
| *mmu-Myog* | AATGCACTGGAGTTCGGTCC | TTCGTCTGGGAAGGCAACAG |
| *mmu-Myf5* | TCTGGTCCCGAAAGAACAGC | GCTCGGATGGCTCTGTAGAC |
| *mmu-Myh1* | CGGGAAGACTGTGAACACGA | TGATTTGGTCCTCCAGCGTC |
| *mmu-Myh2* | GGAAGTCCGAAAAGGAGCGA | GCTCCTGCTTCTGTTTTCACA |
| *mmu-Myh4* | TGAACTGTCAACCCAGAAGGC | TTCATACTGTTCCCGCAGCA |
| *mmu-Myh7* | ACTGTCAACACTAAGAGGGTCA | TTGGATGATTTGATCTTCCAGGG |
| *mmu-FBXO32* | CGTGAGCGACCTCAGCAGTT | TATCAGCTCCAACAGCCGGA |
| *mmu-TRIM63* | GATTCCCGTCGAGTGACCAA | ATGGCAGTTTCCACCAGCTT |
| *hsa-MYOMERGER* | TGGGCTGTCTGCTGTTCATT | CACTTTTGGGGGCCTAACCT |
| *has-MYOMAKER* | CGACGAACCCAAGAGGTCAA | CACTTTGCCGCGATGATGAG |
| *hsa-TNNI2* | GGAGCTGGAGAAGGAGGAGA | CTCTTCAGCCGCATCGATCT |
| *hsa-MYOD* | GCCACAACGGACGACTTCTA | AGTGCTCTTCGGGTTTCAGG |
| *hsa-MYOG* | CAGGGGATCATCTGCTCACG | GGAAGGCCACAGACACATCT |
| *hsa-MYF5* | TCTGGTCCCGAAAGAACAGC | GCTCGGATGGCTCTGTAGAC |
| *hsa-MYH1* | AGCTGAGGTGTAACGGTGTG | TCTAGGAGCCCCAGAAGACC |
| *hsa-MYH2* | CTGAGGTGTAACGGTGTGCT | AAGACCTTGGTGTGCCCAAA |
| *hsa-MYH4* | TGGAGCATGAGCTTGTCCTG | AGGATTCTGCTTGGGAAGCC |
| *hsa-MYH7* | TGACGTCACCTCCAACATGG | TTGACAGAACGCTGGGTCTC |
